# Atrial cardiomyocytes contribute to the inflammatory status associated with atrial fibrillation in right heart disease

**DOI:** 10.1093/europace/euae082

**Published:** 2024-03-28

**Authors:** Ewen Le Quilliec, Charles-Alexandre LeBlanc, Orlane Neuilly, Jiening Xiao, Rim Younes, Yasemin Altuntas, Feng Xiong, Patrice Naud, Louis Villeneuve, Martin G Sirois, Jean-François Tanguay, Jean-Claude Tardif, Roddy Hiram

**Affiliations:** Department of Medicine, Montreal Heart Institute, University of Montreal, 5000 Belanger Street, Montreal, QC HIT 1C8, Canada; Department of Medicine, Montreal Heart Institute, University of Montreal, 5000 Belanger Street, Montreal, QC HIT 1C8, Canada; Department of Medicine, Montreal Heart Institute, University of Montreal, 5000 Belanger Street, Montreal, QC HIT 1C8, Canada; Department of Medicine, Montreal Heart Institute, University of Montreal, 5000 Belanger Street, Montreal, QC HIT 1C8, Canada; Department of Medicine, Montreal Heart Institute, University of Montreal, 5000 Belanger Street, Montreal, QC HIT 1C8, Canada; Department of Medicine, Montreal Heart Institute, University of Montreal, 5000 Belanger Street, Montreal, QC HIT 1C8, Canada; Department of Medicine, Montreal Heart Institute, University of Montreal, 5000 Belanger Street, Montreal, QC HIT 1C8, Canada; Department of Medicine, Montreal Heart Institute, University of Montreal, 5000 Belanger Street, Montreal, QC HIT 1C8, Canada; Department of Medicine, Montreal Heart Institute, University of Montreal, 5000 Belanger Street, Montreal, QC HIT 1C8, Canada; Department of Medicine, Montreal Heart Institute, University of Montreal, 5000 Belanger Street, Montreal, QC HIT 1C8, Canada; Department of Medicine, Montreal Heart Institute, University of Montreal, 5000 Belanger Street, Montreal, QC HIT 1C8, Canada; Department of Medicine, Montreal Heart Institute, University of Montreal, 5000 Belanger Street, Montreal, QC HIT 1C8, Canada; Department of Medicine, Montreal Heart Institute, University of Montreal, 5000 Belanger Street, Montreal, QC HIT 1C8, Canada

**Keywords:** Right heart disease, Cardiomyocyte, Inflammation, Fibrosis, Atrial fibrillation

## Abstract

**Aims:**

Right heart disease (RHD), characterized by right ventricular (RV) and atrial (RA) hypertrophy, and cardiomyocytes’ (CM) dysfunctions have been described to be associated with the incidence of atrial fibrillation (AF). Right heart disease and AF have in common, an inflammatory status, but the mechanisms relating RHD, inflammation, and AF remain unclear. We hypothesized that right heart disease generates electrophysiological and morphological remodelling affecting the CM, leading to atrial inflammation and increased AF susceptibility.

**Methods and results:**

Pulmonary artery banding (PAB) was surgically performed (except for sham) on male Wistar rats (225–275 g) to provoke an RHD. Twenty-one days (D21) post-surgery, all rats underwent echocardiography and electrophysiological studies (EPS). Optical mapping was performed *in situ*, on Langendorff-perfused hearts. The contractility of freshly isolated CM was evaluated and recorded during 1 Hz pacing *in vitro*. Histological analyses were performed on formalin-fixed RA to assess myocardial fibrosis, connexin-43 levels, and CM morphology. Right atrial levels of selected genes and proteins were obtained by qPCR and Western blot, respectively. Pulmonary artery banding induced severe RHD identified by RV and RA hypertrophy. Pulmonary artery banding rats were significantly more susceptible to AF than sham. Compared to sham RA CM from PAB rats were significantly elongated and hypercontractile. Right atrial CM from PAB animals showed significant augmentation of mRNA and protein levels of pro-inflammatory interleukin (IL)-6 and IL1β. Sarcoplasmic–endoplasmic reticulum Ca^2+^-ATPase-2a (SERCA2a) and junctophilin-2 were decreased in RA CM from PAB compared to sham rats.

**Conclusions:**

Right heart disease-induced arrhythmogenicity may occur due to dysfunctional SERCA2a and inflammatory signalling generated from injured RA CM, which leads to an increased risk of AF.

## Graphical Abstract

**Graphical abstract:**
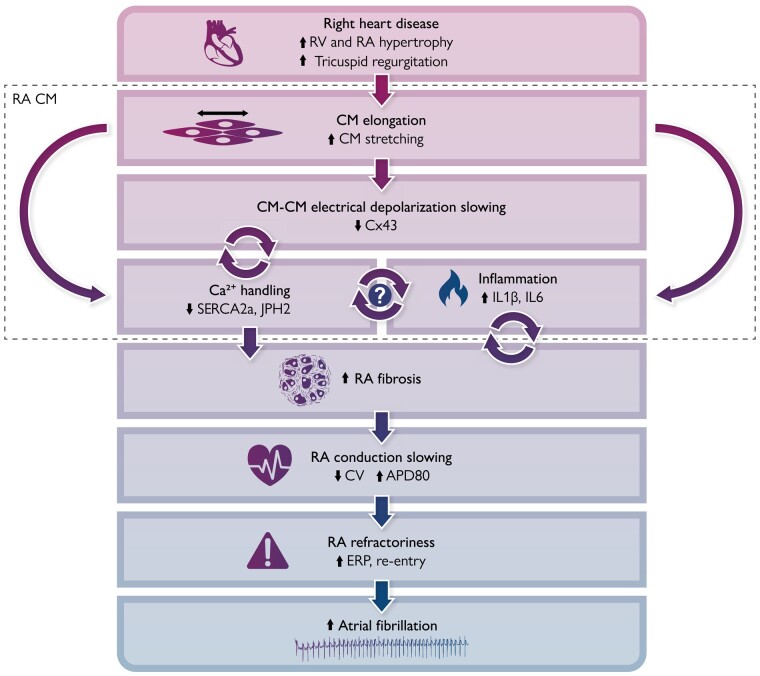
Conditions associated with an increased right-sided cardiac pressure–volume overload can lead to right ventricular (RV) and right atrial (RA) dilation and hypertrophy accompanied by tricuspid valve regurgitation. Chronic dilation of RV and RA chambers provokes mechanical stretch of the cardiomyocytes (CM). Stretched RA CM show signs of dysfunctional gap-junctions, including connexin-43, involved in the propagation of the electrical depolarization. Persistent RA CM stretch is an abnormal condition that may promote dysfunctional Ca^2+^-handling and activate the inflammatory response. It is unclear whether inflammation can also be a cause or a consequence of abnormal Ca^2+^-handling. However, evidence suggests that sustained CM contractile disturbance and chronic inflammation are promotors of atrial fibrosis. Right atrial fibrosis is a physical barrier provoking conduction slowing and promoting electrical re-entry circuits. Altogether, these phenomena occurring in the RA during right heart disease (RHD) constitute an arrhythmogenic substrate increasing the vulnerability to AF. Red arrows: increased (up) or decrease (down) parameter in RHD. Dashed square: Events occurring in the RA CM during RHD.

**Translational perspective**

Our findings suggest that constriction of the pulmonary artery trunk can generate right-sided cardiac hypertrophy, right atrial (RA) dilation, RA inflammation, and fibrosis leading to an increased risk of atrial fibrillation. Such right-sided heart remodelling affects the RA contractility by increasing RA cardiomyocytes’ (CM) length, decreasing the RA CM expression of sarcoplasmic/endoplasmic reticulum Ca^2+^-ATPase 2a, and increasing the RA CM expression of pro-inflammatory interleukins IL6 and IL1β.

## Introduction

Right heart disease (RHD) and right heart failure can be caused by pathologies responsible for the chronic augmentation of right ventricular (RV) blood-volume and -pressure overload.^1,2^ Chronic thrombo-embolic pulmonary hypertension (PH), pulmonary artery hypertension (PAH), pulmonary trunk stenosis, or tetralogy of Fallot have been designated among the important risk factors of RHD.^2,3^ Mounting evidence suggests that cardiac arrhythmias including ventricular and atrial fibrillation (VF and AF) are serious complications of RHD.^2,4–6^

A recent clinical trial revealed that in patients with chronic thrombo-embolic pulmonary hypertension, the prevalence of atrial arrhythmias including AF and atrial flutter (AFl) was 27%.^7^ In a retrospective study involving patients with PAH, 10% of patients showed episodes of supraventricular arrhythmias (SVA). Among those, 42.8% had AF and 82% of SVA episodes were caused by clinical worsening of RHD.^8^ Moreover, studies have suggested that AF is commonly observed in COPD patients in clinical practice. In a meta-analysis totalizing 4.2 million AF patients, the prevalence of COPD was 13%.^9^ The Rotterdam Study has shown a 28% increased risk of AF in patients with COPD.^10^ The pathophysiology underlying the association between AF and COPD remains poorly described, and whether COPD is a cause or consequence of AF remains unclear. In addition, a study of the MACHD (Mayo Adult Congenital Heart Disease) database involving 415 patients with tetralogy of Fallot has shown that 21% had AF.^11^ In this study, AF was considered an important risk factor for heart failure hospitalization and mortality.^11^ Altogether, these reports suggest that RHD provokes an arrhythmogenic substrate affecting the atrium, which leads to AF.

Our recent investigations highlighted that inflammation might be an important contributor to the pathophysiological relationship between RHD and AF.^4,12^ However, our precedent model of monocrotaline (MCT)-induced RHD was a limited approach to studying the suspected RHF-associated atrial inflammation.^4,12^ Monocrotaline is a pneumo-toxic substance that causes severe pulmonary inflammation.^13^ Hence, it is unclear whether the cardiac inflammation observed in MCT-induced RHD is due to (i) right-sided remodelling alone or (ii) the circulating inflammatory agents generated from the injured lung.^4,12^ To avoid this issue, we studied AF incidence in a model of RHD induced by pulmonary artery trunk banding (PAB).^14^

We hypothesized that PAB-induced RHD leads to right-sided cardiac dysfunctions affecting the right atrial (RA) cardiomyocytes (CM), leading to CM-orchestrated inflammation and AF.

The main objectives of this study were to: (i) evaluate AF inducibility in a rat model of PAB-induced RHD; (ii) describe whether RA CM contractility is perturbated by PAB-induced RHD; (iii) determine whether RA CM are involved in the atrial inflammatory response to volume/pressure overload-induced RHD.

## Methods

### Animal groups and pulmonary artery banding surgery

**Figure 1:**
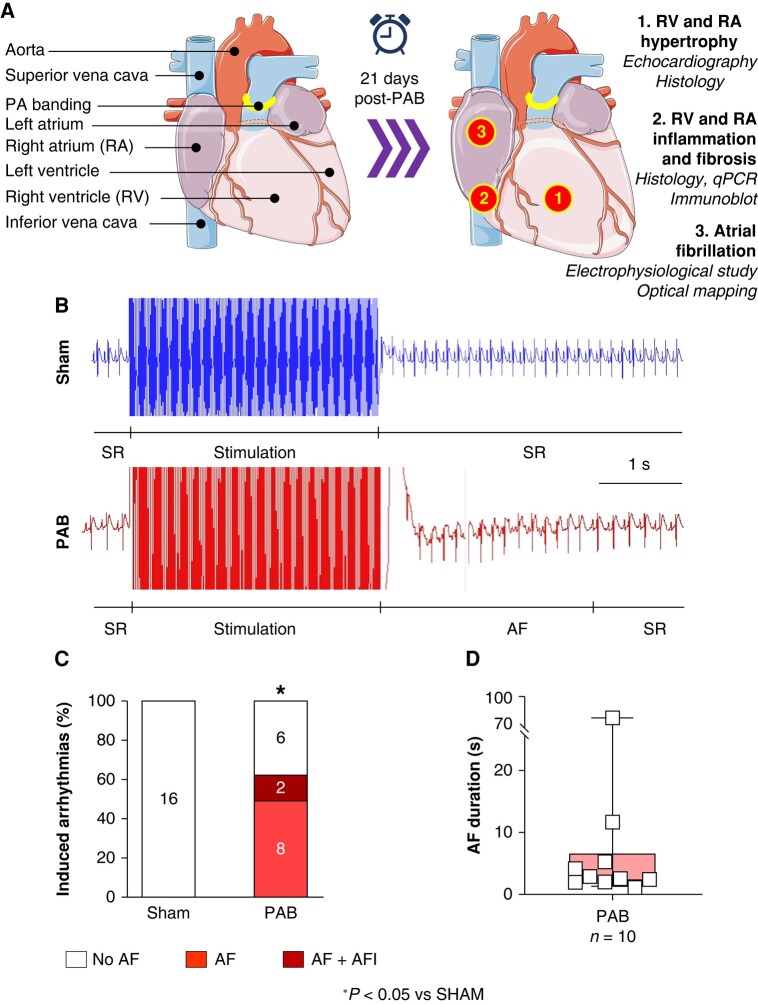
Atrial fibrillation (AF) vulnerability. (*A*) Schematic of a pulmonary artery (PA) trunk banding (PAB) and the methods employed to analyse the right-sided remodelling that occurred 3 weeks post-PAB (*B*) Representative ECGs showing sinus rhythm (SR), 3 s burst stimulation, and post-induction SR or induced AF episode in sham or PAB rats. (*C*) Inducibility of AF and AFl during transesophageal EPS *in vivo*. (*D*) Mean duration of induced AF in inducible PAB rats. (Statistical analysis *C*: Fisher’s exact test. *D*: Each point results from an individual animal.)

Study design and procedures were approved by the Animal Ethics Committee of the Montreal Heart Institute, in accordance with the Canadian Council on Animal Care and the NIH Guide for the Care and Use of Laboratory Animals (protocol number: 2021-2938:2021-47-01). Adult male Wistar rats weighing 225–275 g (Charles River Laboratories, Montreal, QC, Canada), were randomized to two groups: sham and PAB (see Supplementary material online, *Figure S1*). At Day 0 (D0), all PAB animals were anaesthetized with 2–3% isoflurane and 2 L/min O_2_. A dose of 0.05 mg/kg buprenorphine was then administrated subcutaneously for analgesia. The thorax was shaved and disinfected with 2% chlorhexidine and 70% alcohol. An incision was made at the 3rd and 4th left intercostal area. The pulmonary artery (PA) trunk was then identified and sutured using a Perma-Hand 5-0 silk ligated around a 19G needle used as a lead to lead an arterial diameter of 1 mm (*Figure 1A*). Sham animals received the same procedure without suture of the PA trunk. Animals were housed at the MHI animal facility for 21 days with *ad libitum* access to water and food. On D21 post-PAB, echocardiography, and transoesophageal electrophysiological studies (EPSs) were performed *in vivo*, under 2%-isoflurane anaesthesia. While under continued isoflurane anaesthesia, rats were euthanized by exsanguination. The excised heart was perfused under the Langendorff system for *in situ* optical mapping. Atrial tissues were dissected, snap-frozen, and kept in liquid nitrogen (N_2_) or formalin-fixed and paraffin-embedded for *in vitro*, histological, and biochemical analyses.

All experiments and analyses were performed blinded to experimental groups.

### Haemodynamic

At D21 post-PAB, a 2Fr pressure-transducer catheter [Science P catheter-RAT (ADInstrument Inc., Colorado Springs, CO, USA)] introduced via the right jugular vein was used to measure RA and RV blood pressure, and in the left carotid to determine the left ventricle (LV) and left atrium (LA) blood pressure expressed in millimetres of mercury (mmHg).

### Echocardiography

All rats underwent echocardiography. On D21, animals were anaesthetized under 2%-isoflurane. Phased array 10S probe (4.5–11.5 MHz) was used in a Vivid 7 Dimension system (GE Healthcare Ultrasound, Horten, Norway) to acquire echocardiograms as previously described.^12^ Echocardiography was used to determine the atrial and ventricular dimensions, volume, and function. Among the parameters evaluated, we report the PA peak velocity, the right ventricle (RV) anterior wall dimension at end of diastole (RVAWd), the right ventricle diameter at the end of diastole (RVDd), the tricuspid annulus plane systolic excursion (TAPSE), tricuspid regurgitation, the right atrium diameter at the end of systole (RADs), and also left heart parameters including left ventricle diameter at the end of diastole (LVDd), and left atrial dimension at the end of systole (LADs).

### Transoesophageal stimulation

Electrophysiological studies (EPS) were performed *in vivo* at D21 post-PAB to determine the vulnerability to cardiac arrhythmias with a transoesophageal 4Fr quadripolar catheter (2-mm interpolar distance; St. Jude Medical #401993). Before stimulation, ECG variables, including R–R interval, P-wave duration, PR interval, QRS duration, and QT interval, were measured at rest, during sinus rhythm, on 10 non-subsequent beats. The average value for each ECG variable was represented as one dot per animal. For transoesophageal stimulation, twelve pacing bursts (50-Hz 3-s) were applied (4×-threshold current, 2-ms pulse width), separated by 2-s rest periods.^4^ If an episode of arrhythmia was induced after a burst the next stimulation was not applied to allow identification, observation, and description of the arrhythmia’s type and duration. Atrial fibrillation was defined as an ultrarapid irregular atrial rhythm (>800 b.p.m.). Atrial flutter (AFl) was defined as a regular atrial tachyarrhythmia (600 and 800 b.p.m.). The duration of AF was calculated as the mean duration of all AF episodes induced by catheter stimulation. Iox2 software (version 2.8.0.13, EMKA technologies, Paris, France) was used to acquire and analyse the ECG and the catheter signals.

### Optical mapping

On D21 post-PAB, rats were anaesthetised using 2–3% isoflurane inhalation. The heart was excised and Langendorff-perfused via the aorta with Krebs solution (10 mL/min; 37°C; 95% O_2_; pH 7.5). A period of equilibration of 20 min was performed, then blebbistatin (15-µM) was administered and Di-4-ANEPPS (10 µM. 0.1 mL) injected into the circulating Krebs solution. A pair of bipolar electrodes was meticulously positioned on the superior RA appendage, with the pacing stimuli configured to a 2-ms pulse width and a square-wave current amplitude set at 1.5 times the threshold for regular pacing. The stimulator employed a current stimulus, offering a range of amplitudes spanning from 2 to 10 mA. Right atrial fluorescence signals were obtained at 1–2 kHz with a charge-coupled camera (CardioCCD, RedShirtImaging, Decatur, GA, USA) focused on an 10 × 10 mm region of the tissue free wall. The RA effective refractory period (ERP) was determined by S1–S2 protocol where a series of seven S1 pacing at basic CL (BCL) of 150 ms were followed by an S2-extrastimulus, decrementing by 1 ms from 80 ms until failure to capture. Atrial fibrillation inducibility was evaluated on isolated hearts *in situ*, at RA chamber, by applying 25-Hz stimulation. Conduction velocity (CV) and action potential duration to 80% repolarization (APD_80_) were evaluated at BCLs 300, 250, 150, 100, 80, and 60 ms, and calculated with a Matlab® algorithm as previously described.^15^

### Histological analyses and morphometry

Excised RA samples were formalin-fixed and stained with Masson’s trichrome solution to quantify fibrous content at D21 post-PAB. Immunofluorescence was performed on formalin-fixed RA to detect and localize gap-junction connexin-43 (Cx43) using polyclonal antibody anti-Cx43/GJA1 (concentration: 1/100–1/500). CM nucleus was identified using 4-,6-diamidino-2-phenylindole (DAPI) (concentration: 1/1000). Wheat Germ Agglutinin (WGA) membrane staining was used to delineate cell borders, and CM were identified with positive troponin I (1/100). Image Pro Premier 9.3 Software (MediaCybernetics, MD, USA) was used for the detection and quantification of fibrous-tissue content. Confocal microscope (Zeiss LSM 710) to acquire images from immunofluorescence. The presence of Cx43 in sham compared to PAB was reported as the surface occupied by positive Cx43 staining as a function of total atrial surface. Right atrial CM length (µm), width (µm), and surface (µm^2^) were also determined during immunofluorescence analyses.

For each histological analysis described above, five images were captured per histological slide. An observer blinded to group identity quantified the specific parameters of interest. The average value was determined for each slide and animal, then values were compared between sham and PAB.

### Contractility measurement

At D21 post-PAB surgery, RA CM were freshly isolated and analysed in a myocyte contractility system (IonOptix, Westwood, MA, USA). Right atrial CM were field-stimulated via 10-ms square-wave pulses with 1.5 times threshold amplitude. Stimuli were delivered by a stimulator via a platinum bipolar electrode mounted on a micromanipulator. CM were paced at 1 Hz and 15–25 V.

A camera focusing on the cytosolic surface of the CM was used to record and analyse CM shortening with the IonWizard-software sarcomere length algorithm (IonOptix, Westwood, MA, USA). IonWizard Sarcomere Length window was adjusted onto the region of interest to minimize distortions. The baseline value for sarcomere length was established at 1.7 µm or above. Five to ten cells were analysed per rat. The mean cell shortening was measured during 1 min for each cell by analysing 10 beats from five random segments of the shortening recording.

### PCR

Atrial tissue samples were freshly isolated, snap-frozen in liquid-N2, and preserved a −80°C. Homogenization of tissue was performed in RA1 lysis buffer. Extraction of RNAs was performed with the Nucleospin RNA II® Kit (Macherey Nagel, Düren, Germany). Concentration of mRNA was assessed on Nanodrop-2000 (Thermo Fisher Scientific, Watham, MA, USA), and 250 ng was retrotranscribed with the High-Capacity cDNA Reverse Transcription Kit (Thermo Fisher Scientific, Waltham, MA, USA).

RT-PCR was performed using a StepOnePlus Real-Time PCR System (Thermo Fisher). Taqman probes were used for L-type calcium channel (*Cacna1c*), interleukin-1 beta (*Il1b*), interleukin-6 (*Il6*), potassium channel subunit (*Kcnq1*), NOD-like receptor family, and pyrin-domain containing-3 (*Nlrp3*). SYBR-green primers were used for connexin 43 (*Cx43*), phospholamban (*Pln*), ryanodine receptor-2 (*Ryr2*), sodium channel alpha-subunit 5 (*Scn5a*), sarcoplasmic reticulum calcium (Ca^2+^)-ATPase-2a (*Serca2a*), transforming growth factor-beta 1 (*Tgfb1*). Taqman probes and SYBR-green primer sequences (forward and reverse) are provided in Supplementary material online, *Tables S1* and *S2*.

Gene-expression levels were calculated with the 2^−ΔCt^ method and glyceraldehyde-3-phosphate dehydrogenase (*Gapdh*) was the internal standard.

### Immunoblot—rat atrial tissue

Proteins were separated by electrophoresis on 4–15% sodium dodecyl sulphate polyacrylamide gels and transferred onto polyvinylidene difluoride membranes. Membranes were blocked using Tris-buffered saline (TBS) containing 0.2% (volume/volume) Tween-20 and 5% (weight/volume) non-fat milk. Primary antibodies diluted in TBS containing 0.2% Tween-20 were used to incubate the membranes overnight at 4°C. Horseradish peroxidase-conjugated was the secondary antibody. Bands were detected with enhanced chemiluminescence (ECL) and captured with the Bio-Rad ChemiDoc Imaging System.

All expression data were relative to total protein on membrane stained with No-Stain™ Protein Labeling Reagent (Thermo Fisher Scientific, Carlsbad, CA, USA). The images of membranes were captured UV-light transillumination. Image lab software (Bio-Rad, Hercules, CA, USA) was used to quantify protein levels.

### Drugs and reagents

Blebbistatin and di-4-ANEPPS were obtained from Cedarlane (Burlington, Ontario). Troponin-I was obtained from Abcam (Cambridge, UK). CACNA1C, CX43, IL1β, IL6, KCNQ1, NLRP3, PLN, RYR2, SCN5A, TGFβ1, and GAPDH qPCR probes as well as DAPI and WGA were obtained from Invitrogen (Waltham, MA, USA).

Primary antibodies for immunoblot experiments included: BIN1 (Novus Biologicals, NBP1-89102), CACNA1C (Alomone labs ACC-003), CAV (Novus Biologicals, NBP3-16503), IL6 (ThermoFisher ARC0962), JPH2 (ThermoFisher, PA5-141187), IL1β (ThermoFisher PA5-46956), KCNQ1 (NeuroMab clone N374/10), NLRP3 (Novus Biologicals NBP1-77080SS), SCN5A (Alomone labs ASC-005), RYR2 (Thermofisher MA3-916), SERCA2a (Thermofisher MA3-910).

### Statistical analysis

Distributions’ normality was assessed using Shapiro–Wilk tests. For datasets demonstrating normal distribution, comparisons between two groups were conducted using non-paired Student's *t*-tests. Non-normally distributed continuous data or data for which normality could not be assessed were tested by the Mann–Whitney non-paired non-parametric test. Categorical variables like AF inducibility were analysed by Fisher’s exact test. Multiple-group comparisons were performed using one-way or two-way analysis of variance (ANOVA) followed by *post hoc* Tukey or Bonferroni-corrected Student’s *t*-tests when ANOVA revealed significant group effects. Results are presented as box-and-whisker plots to show all points (individual animal) and outliers, the mean (cross [+] symbol), the median (horizontal line inside the box), the first quartile (Q1: lower side of the box), the third quartile (Q3: upper side of the box), the minimum value (segment below the box), and the maximum value (segment above the box). Statistical significance was defined as two-tailed *P*-values <0.05.

## Results

### Effect of pulmonary artery banding on atrial fibrillation inducibility and ECG modifications *in vivo*

**Figure 2:**
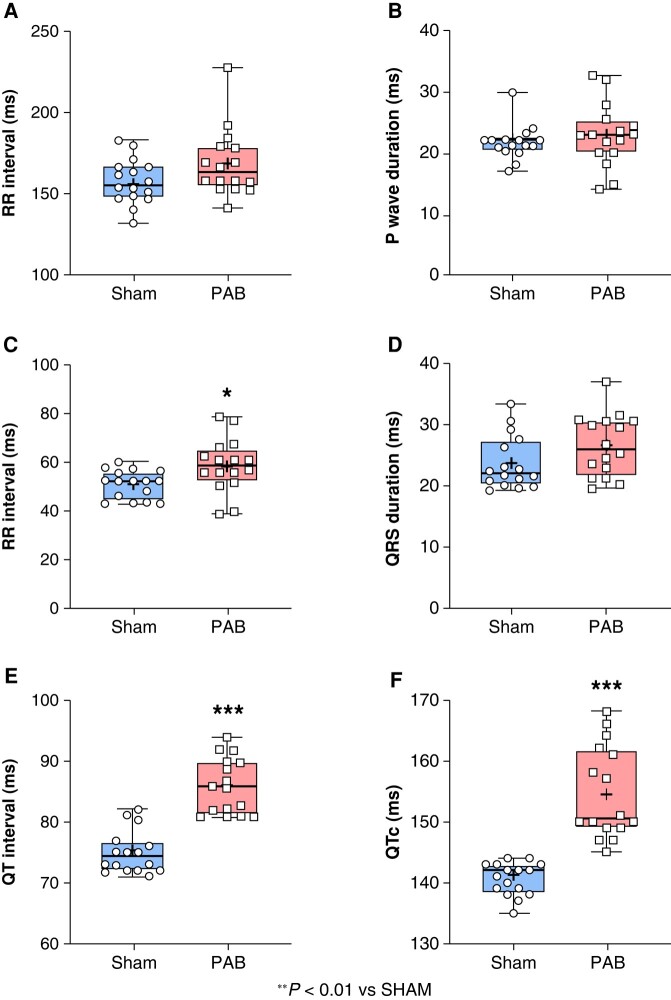
Electrocardiogram analyses. ECG parameters (in ms) were measured, including (*A*) R–R interval, (*B*) P-wave duration, (*C*) P–R interval, (*D*) QRS duration, (*E*) QT interval, and (*F*) QTc. (Statistical analysis: *A*, *B*, *E*, and *F*: non-normal distribution assessed by Shapiro–Wilk test, and statistical difference between groups analysed by Mann–Whitney test. *C* and *D*: Normal distribution assessed by Shapiro–Wilk test, and statistical difference between groups analysed by Student’s *t*-test. Each point represents an individual animal. *n* = 16 rats per group.)

Pulmonary artery banding rats (10/16) were significantly more vulnerable to AF compared to sham animals (0/16) following transoesophageal stimulation *in vivo*. Among PAB animals, 8/16 induced AFl (*Figure* *1B* and *C*). The average AF duration was 11 ± 7 s. ECG variables, including R–R interval, P-wave duration, PR interval, and QRS duration were not statistically significantly different between sham and PAB (*Figure 2A–D*). The QT interval and QTc were significantly increased in PAB rats compared to sham (*Figure* *2E* and *F*).

### Pulmonary artery banding-induced right-sided ventricular structural remodelling

**Figure 3:**
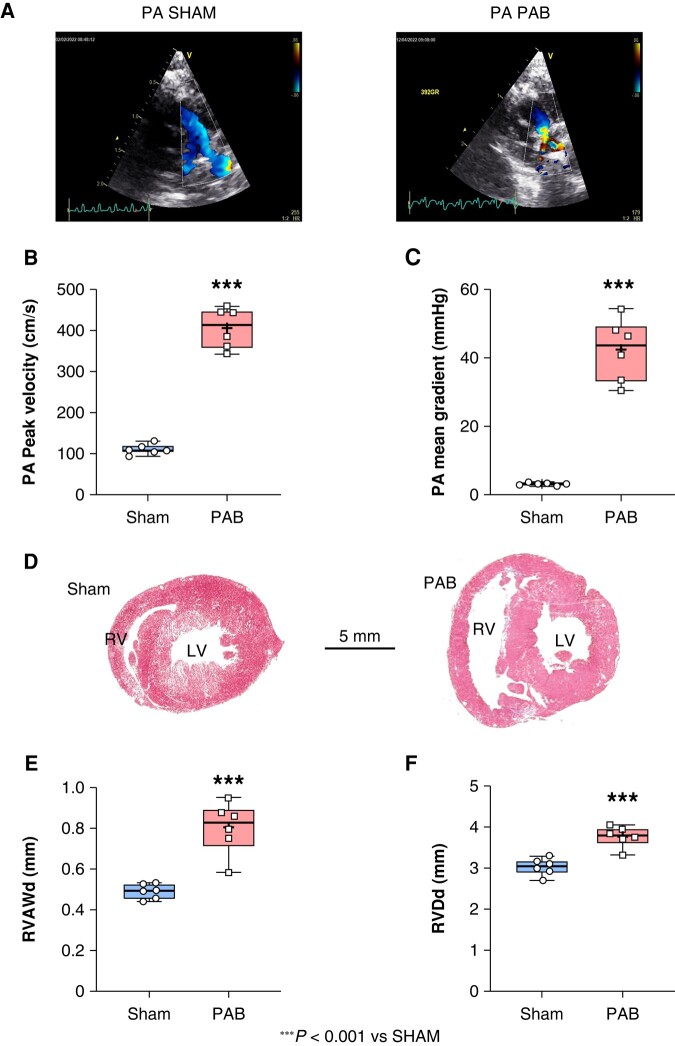
Echocardiography at D21 post-pulmonary artery banding (PAB). (*A*) Echocardiography showing the blood flow through the pulmonary artery (PA) trunk and the left and right PA in sham (left panel) and the reduced flow due to the suture in PAB (right panel). (*B*) Pulmonary artery peak velocity expressed in cm/s and (*C*) PA mean gradient expressed in mmHg in sham and PAB. (*D*) Transverse histological section of the heart, stained by Masson’s trichrome showing the right and left ventricle (RV and LV) in sham (left panel) and PAB (right panel). (*E*) Right ventricle anterior wall at end of diastole expressed (RVAWd) in mm, and (*F*) right ventricle diameter at end of diastole (RVDd) expressed in mm in sham and PAB. (Statistical analysis: Data were normally distributed as assessed by Shapiro–Wilk test and statistical difference between groups was analysed by Student’s *t*-test. Each point represents an individual animal. *n* = 6 rats per group.)

Echocardiography performed on Day 21 post-PAB revealed that the permanent suture provoked a severe reduction of blood flow through the PA (*Figure 3A*) as assessed by a significant 25% increase in PA peak velocity in PAB compared to sham (***P < 0.001) (*Figure 3B*), and a significantly increased PA pressure in PAB vs. sham (***P < 0.001) (*Figure 3C*). Such constriction generated a significant increased blood-volume overload in the RV and the RA, as assessed by haemodynamic measurement (RVSP, RAP) reported in Supplementary material online, *Figure S2*. Chronic RVSP provoked a significant augmentation of RV wall thickness as indicated by a RVAWd of 0.5 ± 0.02 mm in PAB compared to 0.8 ± 0.05 mm sham (***P < 0.001) (*Figure 3E*). The RV chamber was also significantly dilated in PAB compared to sham as characterized by a RVDd of 3.76 ± 0.1 mm in PAB compared to 3.02 ± 0.08 mm in sham (***P < 0.001) (*Figure 3F*). Echocardiography and haemodynamic measurements revealed that the LV and the LA structure were not affected by PAB (see Supplementary material online, *Figures S2B*, *D* and *S3A*, *B*). Hence, for all the data presented below, we focused on structural, functional, and electrophysiological changes affecting the RA in PAB compared to sham.

### Pulmonary artery banding-induced right-sided atrial structural remodelling

**Figure 4:**
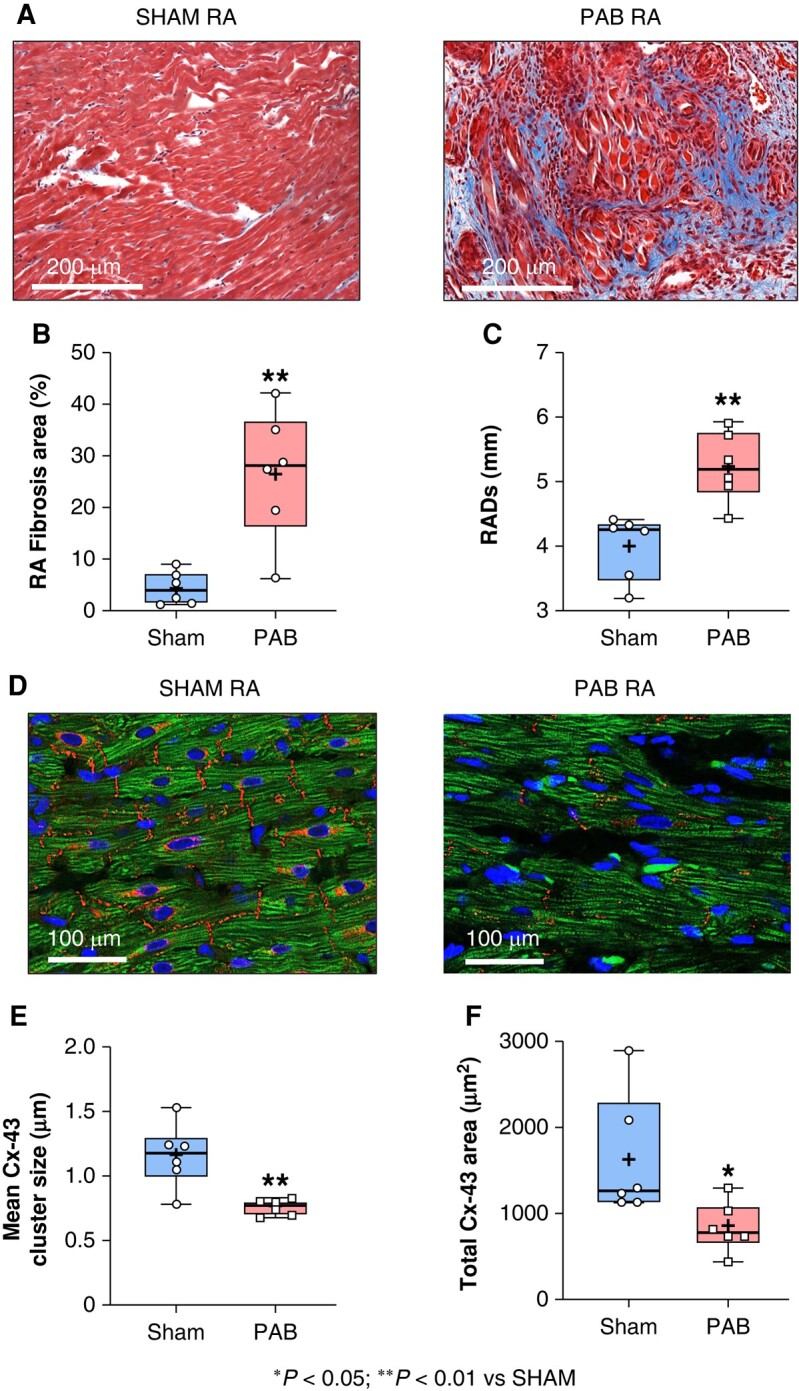
Right atrial fibrosis and gap-junctions. (*A*) Representative transverse histological sections of the right atrium (RA) stained with Masson’s Trichrome solution at D21, in sham (left panel) and pulmonary artery banding (PAB) (right panel). (*B*) Quantification of the RA fibrous content expressed as a percent of the total RA-surface. (*C*) RA dimension at the end of systole (RADs) obtained by echocardiography and expressed in mm in sham and PAB at D21. (*D*) Representative RA tissue following immunofluorescence, showing cardiomyocytes (CM: green), CM nucleus (blue), and connexin-43 (Cx 43: red). (*E*) Average Cx43-cluster size expressed in µm and (*F*) total surface occupied by Cx43 expressed in µm^2^ in sham and PAB. (Statistical analysis: *B*, *C*, and *E*: normal distribution was assessed by Shapiro–Wilk test and statistical difference between the groups was analysed by Student’s *t*-test. *F*: non-normal distribution was assessed by Shapiro–Wilk test and statistical difference between the groups was analysed by Mann–Whitney test. Each point represents an individual animal. *n* = 6 rats per group.)

The PAB-induced RV pressure/volume overload generated significantly increased RA pressure (see Supplementary material online, *Figure S2A*) and RA fibrosis in PAB (26.5 ± 5.1%) compared to sham (4.3 ± 1.3%; **P < 0.01), as assessed by Masson’s trichrome staining (*Figure* *4A* and *B*). Echocardiography revealed a significant enhancement of RADs in PAB (5.3 ± 0.2 mm) compared to sham rats (4.0 ± 0.2 mm; **P < 0.01) (*Figure 4C*). Right atrial dilation was associated with tricuspid valve malfunction as assessed by significantly decreased TAPSE in PAB (1.9 ± 0.1 mm) compared to sham (2.8 ± 0.1 mm; **P < 0.01), reduced tricuspid annulus motion velocity in PAB (0.61 ± 0.05) vs. sham [1.2 ± 0.1; (*P < 0.05)], and development of tricuspid regurgitation in PAB rats (see Supplementary material online, *Figure S3*). Pulmonary artery banding-induced RA dilation was accompanied by gap junctions remodelling (*Figure 4D*). Immunofluorescence revealed that the RA protein expression of Cx43 was significantly decreased in PAB compared to sham (*Figure* *4D*–*F*).

### Pulmonary artery banding-induced right atrial electrophysiological remodelling *in situ*

**Figure 5:**
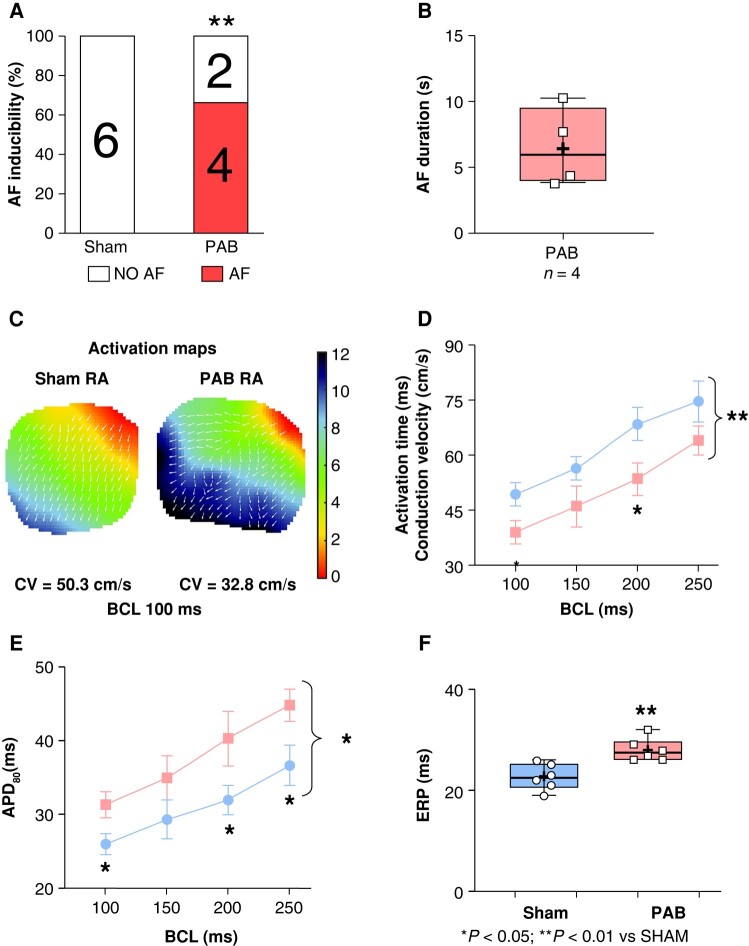
Right atrial optical mapping. (*A*) Inducibility of AF during right atrial (RA) pacing of Langendorff-perfused hearts *in situ*. (*B*) Mean duration of induced AF in inducible pulmonary artery banding (PAB) rats. (*C*) Representative RA activation maps at BCL 100 ms. (*D*) RA conduction velocity at BCL 100, 150, 200, and 250 ms. (*E*) RA action potential duration at 80% repolarization (APD_80_) in sham and PAB rats. (*F*) RA effective refractory period (ERP). (Statistical analysis: *A*: Fisher’s exact test. *B* and *C*: normal distribution assessed by the Shapiro–Wilk test and statistical difference between experimental groups analysed by Student’s *t*-test. *D* and *E*: two-way ANOVA followed by Bonferroni correction. Each point results from an individual animal. *n* = 6 rats per group.)

Consistent with the data obtained *in vivo*, direct burst-pacing of the RA performed *in situ* on freshly isolated and Langendorff-perfused hearts provokes episodes of AF on 4/6 PAB and 0/6 sham rats (*Figure 5A*). The average AF duration was 6.5 ± 1.5 s (*Figure 5B*). The CV was significantly slowed in RA from PAB rats compared to RA from sham (*Figure* *5C* and *D*). The APD_80_ was significantly reduced (*Figure 5E*) and the ERP increased (*Figure 5F*), in RA from PAB animals compared to sham.

### Effect of right-sided heart disease on cardiomyocytes’ morphology

**Figure 6:**
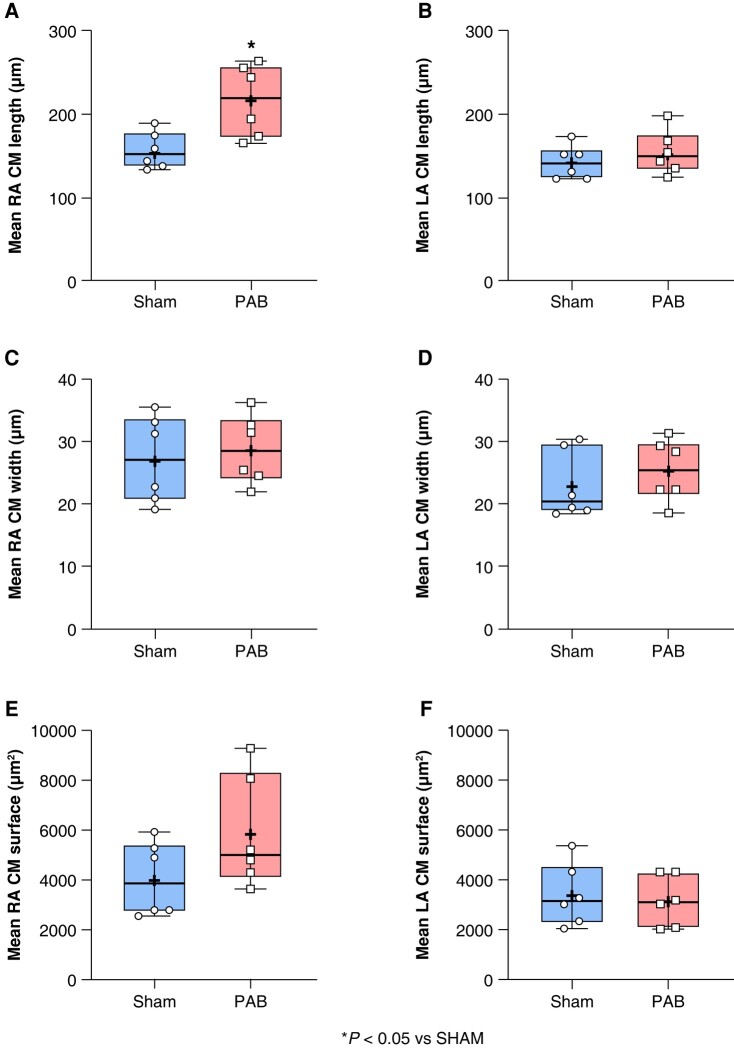
Cardiomyocytes’ (CM) morphometry. Histological slides stained by immunofluorescence were evaluated to determine the CM right and left atrial (RA and LA) length (*A* and *B*), width (*C* and *D*), and surface (*E* and *F*) in sham and pulmonary artery banding (PAB). (Statistical analysis: *A*, *B*, *C*, *E*, and *F*: normal distribution assessed by Shapiro–Wilk test and statistical difference between groups analysed by Student’s *t*-test. *D*: Non-normal distribution assessed by Shapiro–Wilk test and statistical difference between groups analysed by Mann–Whitney test. Each point represents an individual animal. *n* = 6 rats per group.)

The RA and LA were freshly isolated and fixed with 4% formaldehyde, and the atrial CM were stained by immunofluorescence, with WGA as previously reported in *Figure 4D*. Right atrial and LA CM length (*Figure* *6A* and *B*), CM width (*Figure* *6C* and *D*), and CM surface (*Figure* *6E* and *F*) were evaluated. Our data revealed a significant ∼33% augmentation of RA CM length in PAB compared to sham (*Figure 6A*). Left atrial CM morphology was unchanged in PAB compared to sham.

### Effect of right-sided heart disease on cardiomyocytes’ contractility

**Figure 7:**
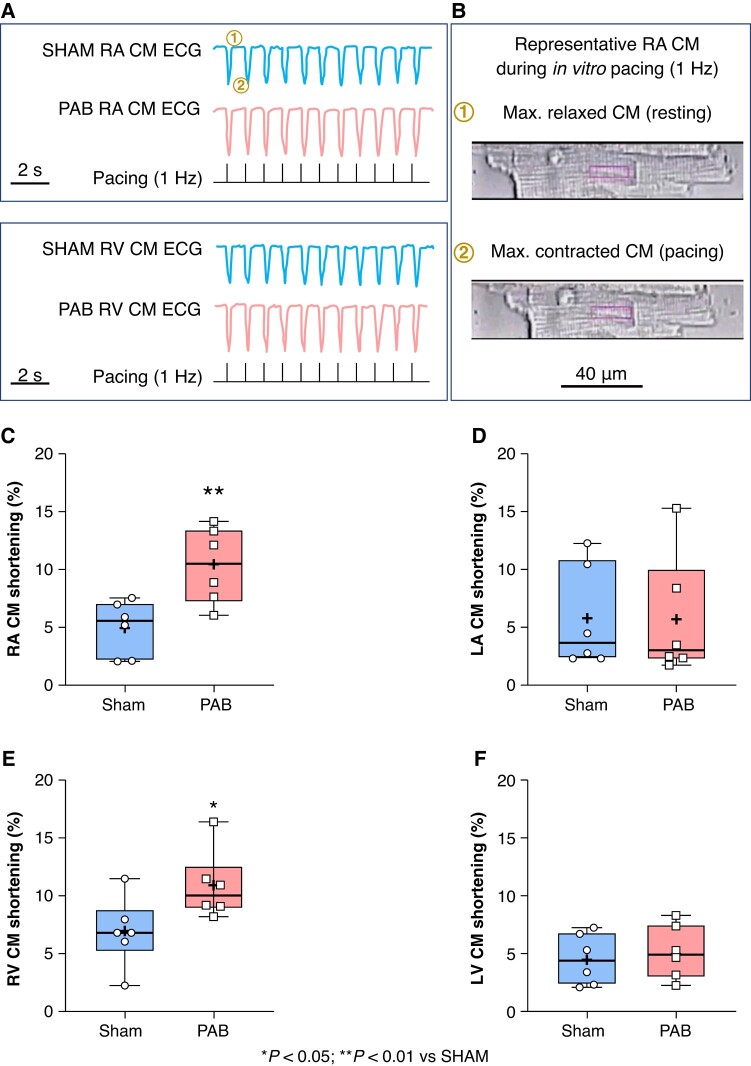
Cardiomyocytes’ (CM) contractility. (*A*) Representative recording of freshly isolated right atrial (RA) and ventricular (RV) CM paced at 1 Hz *in vitro,* from sham and pulmonary artery banding (PAB) rats. (*B*) representative picture of a CM during *in vitro* pacing at maximum relaxation (*A1* and *B1*) and maximum contraction (*A2* and *B2*). Square indicates a random area of focus to measure the CM shortening isolated from the RA (*C*), LA (*D*), RV (*E*), and LV (*F*) in sham and PAB. (Statistical analysis: *C*, *E*, and *F* data were normally distributed as assessed by Shapiro–Wilk test and statistical difference between experimental groups was analysed by Student’s *t*-test; *D*: was non-normally distributed as assessed by Shapiro–Wilk test and statistical difference between experimental groups was analysed by Mann–Whitney test. Each point represents an individual animal. *n* = 6 rats per group.)

Freshly isolated RA, LA, RV, and LV CM were paced at 1 Hz and live recorded (*Figure* *7A* and *B*). Cardiomyocytes length was measured at rest (maximum relaxation state) and at maximum contraction state (*Figure* *7B1* and *B2*). Pulmonary artery banding CM from RA and RV showed significantly more hyper-contractility than respective CM from sham (*Figure* *7C* and *E*). No significant changes were observed in CM from LA and LV in PAB compared to sham (*Figure* *7D* and *F*).

### Effect of pulmonary artery banding-induced right heart disease on the mRNA expression of genes related to calcium-handling machinery, ion channels, and inflammation

**Figure 8:**
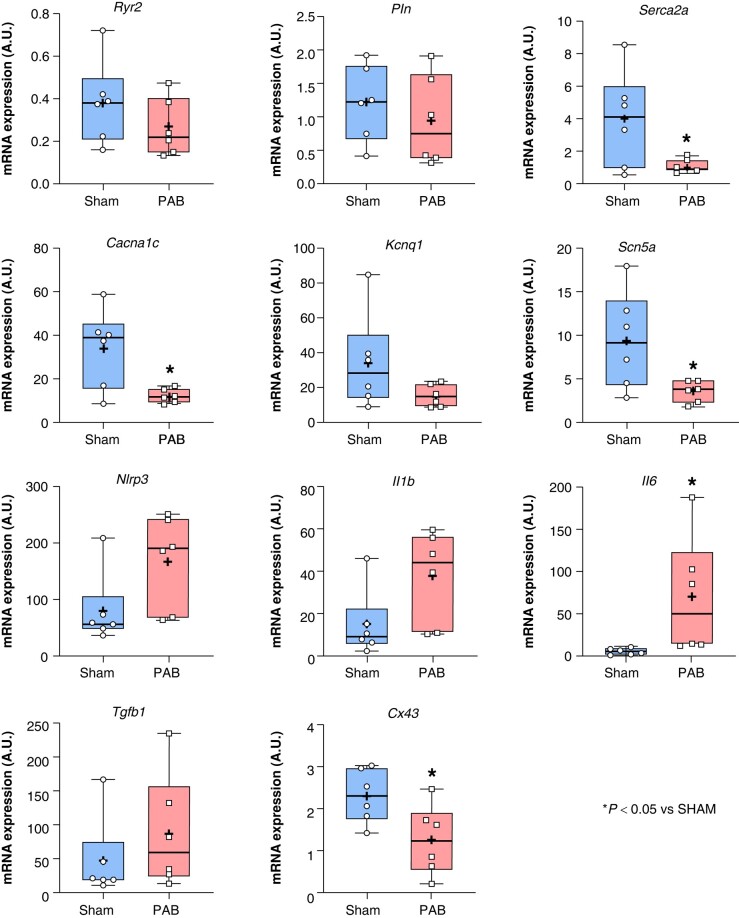
Expression of selected genes. Gene-expression level evaluated by RT-qPCR analysis for calcium-handling-related genes *Ryr2, Pln, Serca,* ion channels-related genes *Cacna1c, Kcnq1, Scn5a*, inflammation-related genes *Nlrp3, Il1b, Il6,* and *Tgfb1,* and connexin *Cx43* in RA from sham and PAB rats. (Statistical analysis: Ryr2, Pln, Serca2a, Cacna1c, Kcnq1, Scn5a, Il6, Cx43 were normally distributed as assessed by Shapiro–Wilk test and statistical difference between the experimental groups was analysed by Student’s *t*-test. Nlrp3, Il1b, and Tgfb1 were non-normally distributed as assessed by Shapiro–Wilk test and statistical difference between the groups was analysed by Mann–Whitney test. Each point represents the level of expression from an individual animal. *n* = 5 rats/group.)

The RA CM mRNA expression of selected genes involved in calcium-handling machinery (*Ryr2, Pln, Serca2*), ion channels (*Cacna1c*, *Kcnq1*, *Scn5a*), inflammation (*Nlrp3, Il1b, Il6, Tgfb1*), and gap-junction (*Cx43*) was evaluated at D21 by qPCR (*Figure 8*). Compared to sham animals, PAB-induced RHD rats showed significantly decreased RA CM expression of *Serca2*, *Cacna1c*, and *Cx43* (*Figure 8*). However, the RA CM expression of *Il6* was significantly increased in PAB compared to sham (*Figure 8*).

### Effect of pulmonary artery banding-induced right heart disease on the expression of selected key proteins

**Figure 9:**
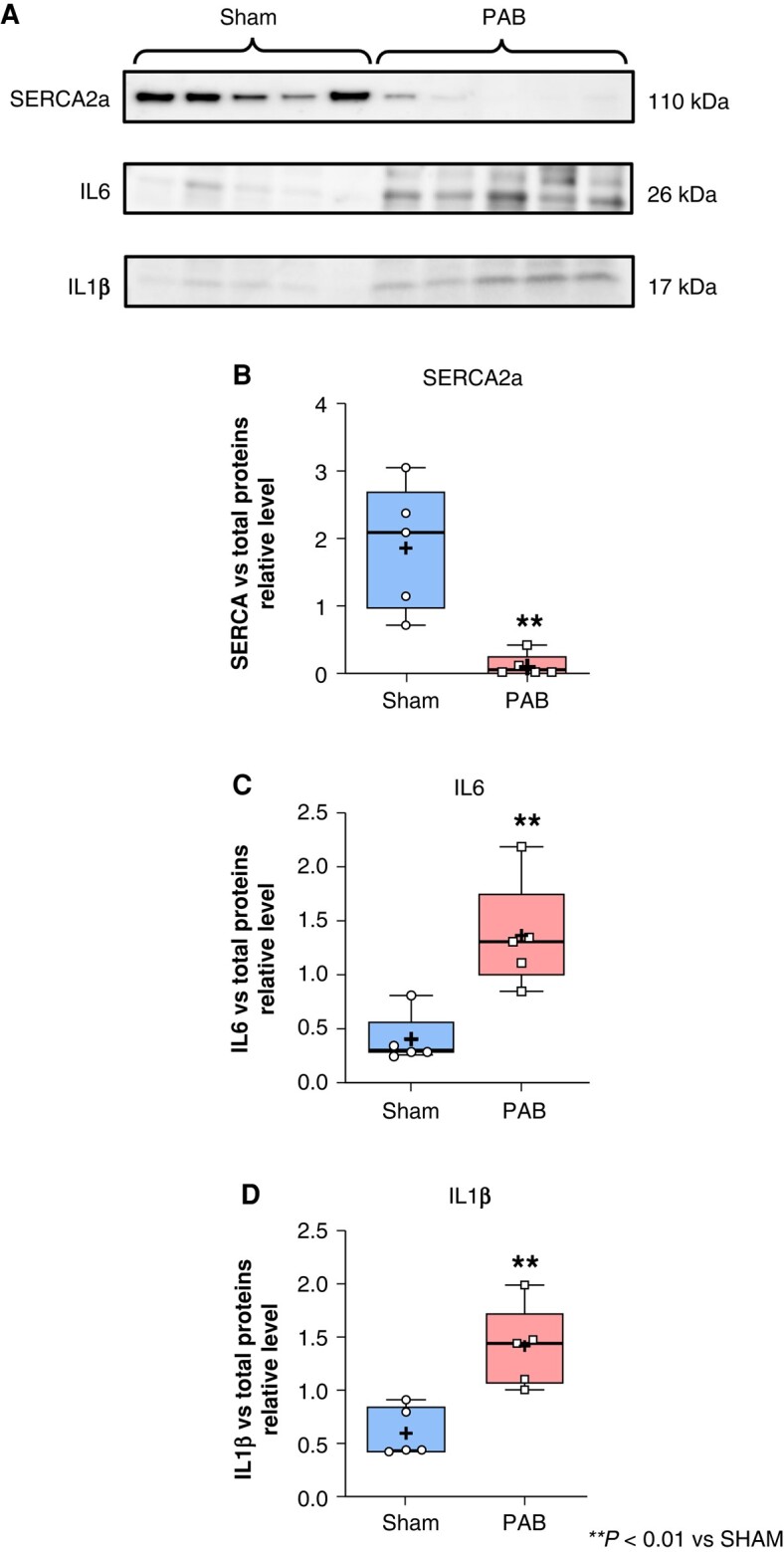
Right atrial expression of SERCA2a, IL6, and IL1β proteins. Protein-expression level evaluated by western blot analysis for SERCA2a (*A* and *B*), IL6 (*A* and *C*), and IL1β (*A* and *D*) in RA from sham and PAB rats. (See uncropped gels and total proteins on blot in Supplementary material online, *Figures S5* and *S6*.) (Statistical analysis: *B* and *D*: normal distribution assessed by Shapiro–Wilk test and statistical difference analysed by Student’s *t*-test. *C*: Non-normal distribution assessed by Shapiro–Wilk test and statistical difference between the groups analysed by Mann–Whitney test. Each point represents the level of expression from an individual animal. *n* = 5 rats/group.)

Right atrial expression of RYR2, SCN5A, CACNA1c, and KCNQ1 was not significantly different in PAB compared to sham (see Supplementary material online, *Figure S4*). However, SERCA2a was significantly decreased (*Figure* *9A*–*C*), whereas IL6 and IL1β were significantly increased in RA from PAB rats compared to sham (*Figure* *9A*–*E*).

**Figure 10:**
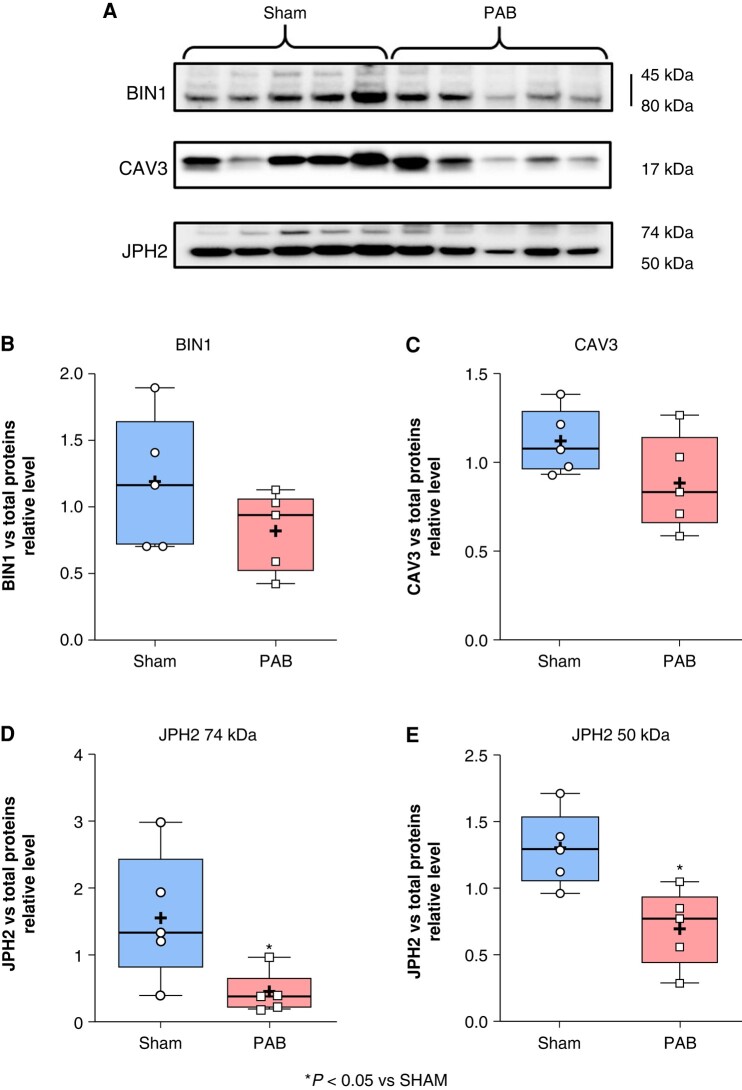
Right atrial expression of BIN1, CAV3, and JPH2 proteins. Protein-expression level evaluated by western blot analysis for BIN1 (*A* and *B*), CAV3 (*A* and *C*), and JPH2 (*A*, *D*, and *E*) in RA from sham and pulmonary artery banding (PAB) rats. (See uncropped gels and total proteins on blot in Supplementary material online, *Figures S11* and *S12*.) (Statistical analysis: data were normally distributed as assessed by Shapiro–Wilk test and statistical difference between the groups was analysed by Student’s *t*-test. Each point represents the level of expression from an individual animal. *n* = 5 rats/group.)

The RA CM transverse tubule function was assessed via the protein expression of BIN1, CAV3, and JPH2. Our data revealed that BIN1 (*Figure* *10A* and *B*) and CAV3 (*Figure* *10A* and *C*) tended to decrease in RA from PAB compared to sham, and JPH2 was significantly reduced in RA from PAB compared to sham (*Figure* *10A, D,* and *E*).

## Discussion

### Main findings

In this study, we found that permanent PAB generated a severe right-sided cardiac remodelling characterized by RV and RA dilation and hypertrophy. Prolonged PA constriction and pressure/volume overload provoked increased CM length and significant CM hyper-contractility in the RV and the RA. Pulmonary artery banding-induced CM over-contraction was accompanied by decreased protein expression of SERCA2a. Such right-sided dysfunctions were associated with RA fibrosis and RA inflammatory profile identified by increased expression of IL6 and IL1β. Compared to sham, RHD-induced RA remodelling led to significant RA conduction slowing and increased AF vulnerability in PAB rats (*Graphical abstract*).

### Pulmonary artery banding-induced cardiomyocyte inflammation and atrial fibrillation

Cardiac fibrosis has been described as a risk factor of AF and mounting evidence suggests that atrial inflammation is also an important contributor of tachyarrhythmia and AF incidence.^16^ In a mice model of depression associated with AF, chronic stress was associated with deregulation of ions involved in atrial electrical conduction (SCN5a, CACNA1C, Cx43) and increased atrial inflammation identified by overexpression of the NACHT, LRR, and PYD domains-containing protein-3 (NLRP3) inflammasome and IL1β.^17^ Recent study revealed that chronic RHD affects the RA CM by provoking CM hypertrophy and RA perivascular fibrosis in patients with PAH.^18^ In our experimental model of PAB-induced RHD, RA CM were also hypertrophied and accompanied with significantly more atrial fibrosis than healthy subjects (*Figure 4*). Atrial fibrosis is known to provoke electrical conduction slowing and re-entry, leading to an increased risk of AF.^19^ However, although some targets (including resolution biomarkers, peptidyl-arginine deiminase, or transglutaminase 2) are proposed in various basic research articles, no available strategy allows an efficient clinical removal of atrial fibrosis to prevent or cure AF.^12,20,21^ Persistent PAB leads to chronic RA volume overload generating a mechanical stretch and stress to the RA myocardium, which may lead to the activation of an inflammatory response in the RA.^2,19^ Recent studies support the idea that prolonged RA remodelling might be associated with the persistence of AF via aggravation of RA inflammation.^22–24^ In our study, mRNA and protein expression of pro-inflammatory interleukins IL6 and IL1β was increased in RA CM from RHD compared to healthy conditions (*Figures 8* and *9*). Studies have shown that patients with AF had significantly higher serum levels of IL1β compared to non-AF patients.^25^ Moreover, IL6 levels have been shown to be elevated in patients with increased atrial size and increased AF duration.^26^ Furthermore, it has been shown that activation of the NLRP3 inflammasome is implicated in the overexpression of IL1β and IL6 in AF.^27–29^ Hence, our data support the idea that RA CM participate in the RA inflammatory status associated with AF in RHD (*Figures 8* and *9*). Future investigations may help to better understand the chronological events and causal link between RA remodelling, inflammation, and AF incidence in RHD.

### Arrhythmogenic interaction between the cardiomyocyte’s calcium-handling machinery and the pro-inflammatory signalling

The regulation of cytoplasmic delivery and removal of calcium (Ca^2+^) is an essential mechanism promoting the correct rate and duration of CM contraction.^30–32^ Atrial CM Ca^2+^-handling machinery is perturbed in RHD as assessed by decreased expression of *Ryr2*, *Pln*, and *Scn5a*.^4^ Recent evidence has contributed to understand that inflammation signalling negatively interacts with the Ca^2+^-handling machinery in a mice model of inflammatory atrial cardiomyopathy, by downregulating *Cacna1c*, Ca^2+^-calmodulin-dependent protein kinase II (*Camk2*), *Ryr2*, and *Serca2*.^33^ Consistent with these results, we recently demonstrated that daily treatment with Resolvin-D1 (RvD1), an inflammation–resolution promoting autacoid, can attenuate Ca^2+^-handling abnormalities by normalizing *Cacna1c*, *Ryr2*, and *Scn5a* expression in AF models of pressure-overload-induced right heart disease, and myocardial infarction.^12,34^

In the present study, we observed evidence of reduced expression of SERCA2a in freshly isolated RA CM from RHD rats compared to sham (*Figures 8* and *9*). This downregulation was associated with increased expression of IL6 and IL1β (*Figures 8* and *9*). Our current study does not elucidate whether the CM inflammation is a consequence or a cause of SERCA2a downregulation in response to RHD, but further investigation may help to clarify this issue by studying RHD-induced atrial remodelling and inflammation at different time points from prior induction of the atrial disease to its severe stages.^19^ Reduction of expression and activity of SERCA2a has recently been demonstrated as an important dysfunction associated with heart failure.^35^ In a rabbit model of chronic kidney disease, Ca^2+^ dysregulation was associated with decreased SERCA2a activity, increased right ventricular outflow tract, and increased incidence of ventricular tachycardia.^36^ Consistent with our current findings suggesting a relationship between cardiac inflammation, SERCA2a downregulation, and cardiac arrhythmias in RHD, it has recently been shown that acute administration of lipopolysaccharide leads to decreased SERCA2a activity and decreased RV strain in a rat model of PAH^37^ (*Graphical abstract*).

These discoveries suggest that novel anti-arrhythmia therapeutic strategies may include a combination of pharmacological approaches targeting SERCA2a or/and inflammation to prevent or cure AF incidence in RHD.^16,38^ Consistent with our findings in RHD presented here, a recently published article reported that pressure overload induced by transverse aortic constriction was responsible for left heart disease (LHD) associated with reduced LA ERP, increased LA inflammation (NF-κB, NLRP3, IL6, TNFa), development of LA fibrosis [characterized by overexpression of collagen (COL)I, COLIII, and TGFβ], and increased risk of AF.^39^ These phenomena were also accompanied by abnormal Ca2^+^-handling as assessed by decreased expression of SERCA2a, and increased RYR2 and PLB.^39^

In the *continuum* of dysfunctions observed in the sarcoplasmic reticulum, the remodelling affecting the transverse tubules (T-tubules) has been suggested as an important event in the pathophysiology of AF.^40^ Studies have shown that deregulation of specific proteins expressed in the T-tubules is associated with arrhythmogenicity and AF.^41^ Downregulation of a mediator of T-tubules function and Ca^2+^-machinery in CMs called bridging integrator 1 (BIN1) has been described to contribute to abnormal cardiac contraction and malignant cardiac arrhythmias in the context of heart failure.^42^ In addition, overexpression as well as downregulation of caveolin-3 (CAV3), a major regulator of T-tubule function, has been shown to be associated with the development of cardiac arrhythmias.^43^ Moreover, the knockdown of junctophilin-2 (JPH2), which is abundant in the T-tubules has been described as interacting with the Ca^2+^-handling machinery via the RYR2 by provoking Ca^2+^-leak from the sarcoplasmic reticulum and inducing increased automaticity and arrhythmogenesis.^44^ Consistent with these observations, in our model of right-sided atrial remodelling, we discovered that JPH2 was significantly decreased in RA from RHD rats compared to sham (*Figure 10*).

### Potential clinical applications of the understanding of cardiomyocytes-orchestrated atrial inflammation in atrial fibrillation management contextualized to right heart disease

The common clinical management of AF includes: (i) the use of anticoagulation for thromboembolism prophylaxis, (ii) strategies of rate control, or (iii) prompt rhythm control.^45^ Rate control implies the use of medications promoting atrioventricular nodal blocking, such as beta-blockers and Ca^2+^-channel blockers, to maintain the normal heart rate.^46^ Rhythm control involves the use of anti-arrhythmic drugs, cardioversion, and catheter ablation to restore and maintain atrial sinus rhythm.^47^ In conditions provoking right-sided cardiac remodelling, AF patients with such RHD often exhibit signs of fragile RV function, which makes it difficult to use rate control agents like beta-blockers or Ca^2+^-channel blockers safely and effectively.^48^ In this context, AF treatment must take into consideration the careful use of combined anti-pressure overload and anti-arrhythmic medications.^2^

In AF patients with PH, radiofrequency catheter ablation (RCA) is used, but is associated with 30–50% of recurrent supraventricular arrhythmias, including AF.^7^ In a clinical study involving 77 patients among which 39 received limited ablation and 38 underwent extensive ablation, PH was associated with RA enlargement and AF, but extensive ablation appeared to not be an optimal approach over limited ablation, to prevent AF recurrence.^49^ Nevertheless, the use of RCA remains a commonly adopted option in the management of AF patients with PH.^50^

Although technically challenging due to RA dilation and tricuspid annulus deformation in PAH, the choice of RCA procedure must also take into consideration that the general anaesthesia required during such procedure is complex and must be employed cautiously, in the context of pulmonary hypertensive patients.^51^

Our current study suggests that novel therapeutic approaches may include inflammation-resolution strategies to prevent or cure the inflammatory status, which is commonly observed in AF and RHD.^2,38,52–55^ In addition, SERCA2a is also dysregulated in both PAH and AF,^56,57^ suggesting that strategies promoting the normal expression and activity of SERCA2a may contribute to preventing AF occurrence in RHD-induced arrhythmogenicity. In a rabbit model of left heart failure, upregulation of SERCA2a was associated with LA cardio-protection and reduction of atrial vulnerability to arrhythmogenicity.^58^ These results support the idea more investigation is required to elucidate the modulatory role of SERCA2a-targeting strategies in AF prevention in the context of RHD.

### Limitations and perspectives

This study suggests that the permanent narrowing of the PA trunk mimicked by our PAB surgery is an effective and reproducible model of RV pressure/volume overload. Although we focused on the expression of IL6 and IL1β, other inflammatory biomarkers could be evaluated in future investigations to better characterize the RA CM inflammatory profile induced by PAB and RHD.

The decreased expression of SERCA2a in RA CM from PAB compared to sham is an interesting avenue to explore in future studies. Pharmacological strategies targeting the inhibition or/and promotion of SERCA2a and its relationship with AF inducibility could be investigated in the context of PAB-induced RHD.

Recent studies suggest that although adult men are more susceptible to AF than women, above 65 years old, women are more likely to develop severe complications of AF such as stroke or sudden death. Hence, in the context of our study, it would be of great interest to include female rats, to evaluate whether differences related to the biological sex exist in terms of PAB-induced AF susceptibility.

To better characterize the impact of inflammation on the incidence of AF in PAB-induced RHD, an anti-inflammatory or pro-resolution approach could be performed to evaluate if the modulation of CM-secreted interleukins can prevent the atrial arrhythmogenic substrate.

## Conclusions

Our results show that permanent PA narrowing by PAB can provoke severe right-sided arrhythmogenic cardiac remodelling including RA dilation, RA fibrosis, and RA conduction slowing. Such remodelling affects the right atrial CM by increasing the cellular length and decreasing the expression of Cx43 and SERCA2a. In addition, PAB-induced RHD is associated with increased expression of pro-inflammatory interleukins IL6 and IL1β. Altogether, these phenomena may have contributed to the observation that PAB-induced RHD provoked an increased vulnerability of AF.

## Supplementary Material

euae082_Supplementary_Data

## Data Availability

All raw data supporting the findings from this study are available from the corresponding authors upon reasonable request.
