# Supplementary material for: Atrial cardiomyocytes contribute to the inflammatory status associated with atrial fibrillation in right heart disease
Source: Europace. 2024 Mar 28;26(4):euae082. doi: 10.1093/europace/euae082 (PMC11000822; doi:10.1093/europace/euae082)
Supplement: euae082_Supplementary_Data [file euae082_supplementary_data.pdf]

SUPPLEMENTARY MATERIAL ONLINE TABLES AND FIGURES

Atrial Cardiomyocytes contribute to the Inflammatory Status associated with Atrial Fibrillation in Right Heart Disease.

Ewen Le Quilliec[1], Charles-Alexandre LeBlanc[1], Orlane Neuilly[1], Jiening Xiao[1], Rim Younes[1], Yasemin Altuntas[1], Feng Xiong[1], Patrice Naud[1], Louis Villeneuve[1], Martin G. Sirois[1], Jean-François Tanguay[1], Jean-Claude Tardif[1], and Roddy Hiram[1,*]

[1]Montreal Heart Institute, Department of Medicine, University of Montreal, Montreal, Canada.

***Address for Correspondence:** Dr. Roddy Hiram, PhD, Montreal Heart Institute Research Center, 5000 Belanger Street Montreal, Quebec. Canada H1T 1C8

Tel: 514-376-3330 ext.: 5015

Email: roddy.hiram@icm-mhi.org

Table of Contents

SUPPLEMENTARY MATERIAL ONLINE TABLES and FIGURES 1
Table S1 Taqman probes 3
Table S2 SYBR green primers 4
Figure S1 Study Design 5
Figure S2 Hemodynamic Measurements. 7
Figure S3 Echocardiography……………………………………….. ……...............…..9
Figure S4 Protein expression of ion channel-related genes 11
Figure S5 SERCA2a Original Western blot gel and Total proteins stained on Blot………….13
Figure S6 IL6 and IL1β Original Western blot gels and Total proteins stained on Blot.. 15
Figure S7 RYR2 Original Western blot gel and Total proteins stained on Blot. 17
Figure S8 SCN5A Original Western blot gel and Total proteins stained on Blot………..…..19
Figure S9 CACNA1c Original Western blot gel and Total proteins stained on Blot. ……..….. 21
Figure S10 KCNQ1 Original Western blot gel and Total proteins stained on Blot. 23
Figure S11 BIN1 Original Western blot gel and Total proteins stained on Blot…………….. 25
Figure S12 CAV3 and JPH2 Original Western blot gel and Total proteins stained on Blot…..27

Gene	Probe
Cacna1c	Rn01492576_m1
Il1b	Rn00580432_m1
Il6	Rn01410330_m1
Kcnq1	Rn00583376_m1
Nlrp3	Rn04244620_m1

Table S1. Taqman probes

Gene	Forward primer	Reverse Primer
Cx43	AGGCGTGAGGAAAGTACCAA	GCACTCCAGTCACCCATGTC
Pln	TGTGACGATCACAGAAGCCAA	CCTGATAGCCGAGCGAGTAAG
Ryr2	CACGGAAATGACATCGCGTC	CTCCGTGTCTATGGGGATGC
Scn5a	CGTCATGGTCATTGGCAACC	TCATCTCCCCATCCTCGTCA
Serca2a	TAAAATAGAACGCGCCAACGC	CTTCTGGAGCACCCTTCACA
Tgfb1	CCATGACATGAACCGACCCT	TGCCGTACACAGCAGTTCTT
Gapdh	GCATCTTCTTGTGCAGTGCC	GAGAAGGCAGCCCTGGTAAC

Table S2. SYBR green primers

Online Figure S1

A Timeline

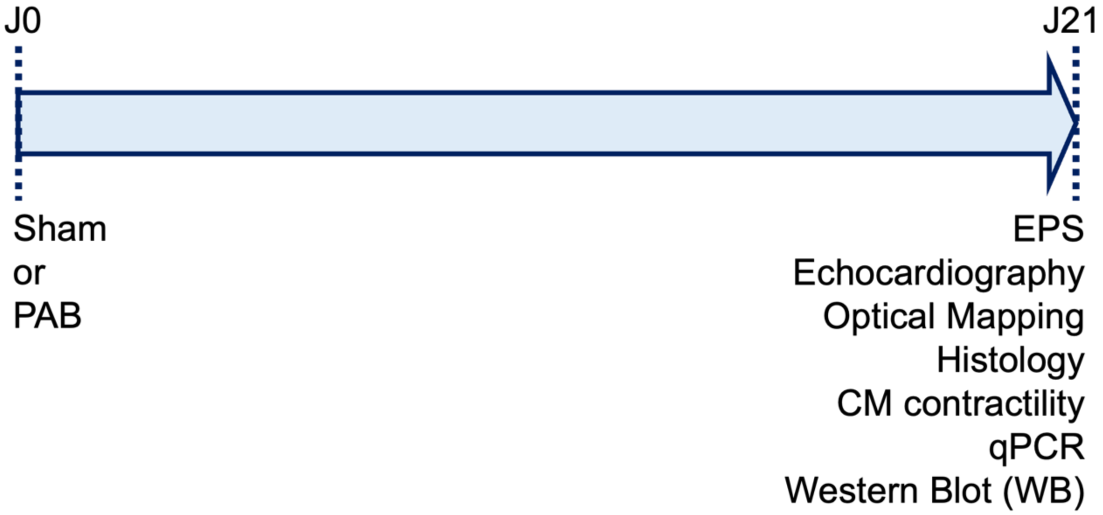

B Distribution of animals

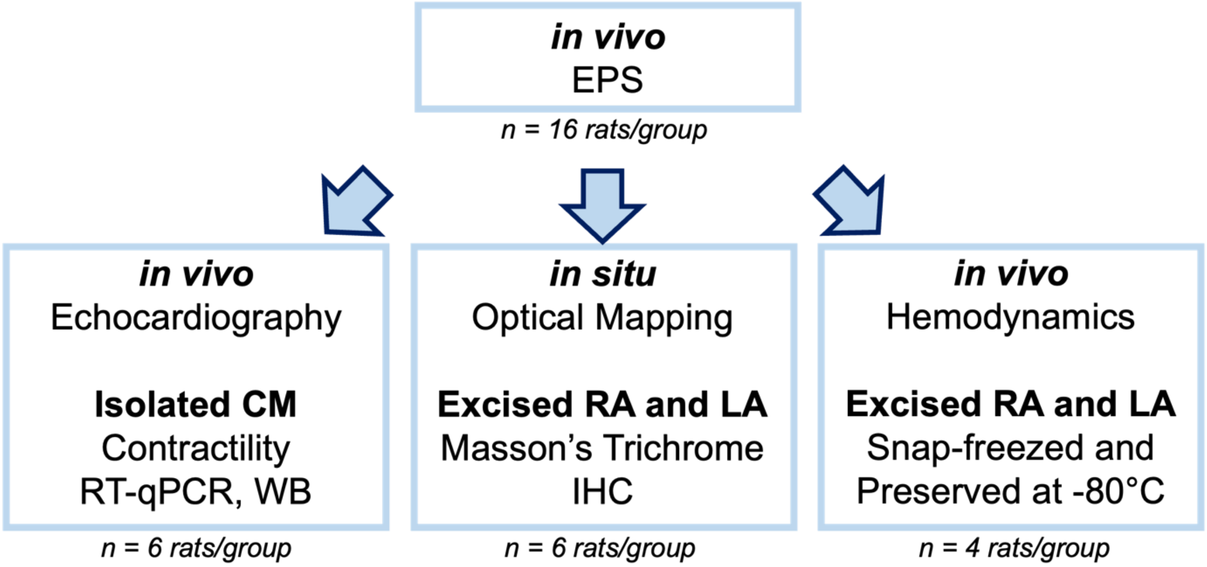

Figure S1. Study design. Schematic of the study design showing (**A**) the chronological timeline, the animal groups (sham and PAB) and the main experiments performed; and (**B**) the distribution of animals for each experiment. All subjects underwent *in vivo* EPS (n = 16 rats per group). Animals were randomly partitioned for remaining *in vivo*, ex-vivo and *in situ* experiments including echocardiography (n = 6 rats per groups), hemodynamics (n = 4 per groups), optical mapping (n = 6 per group).

Online Figure S2

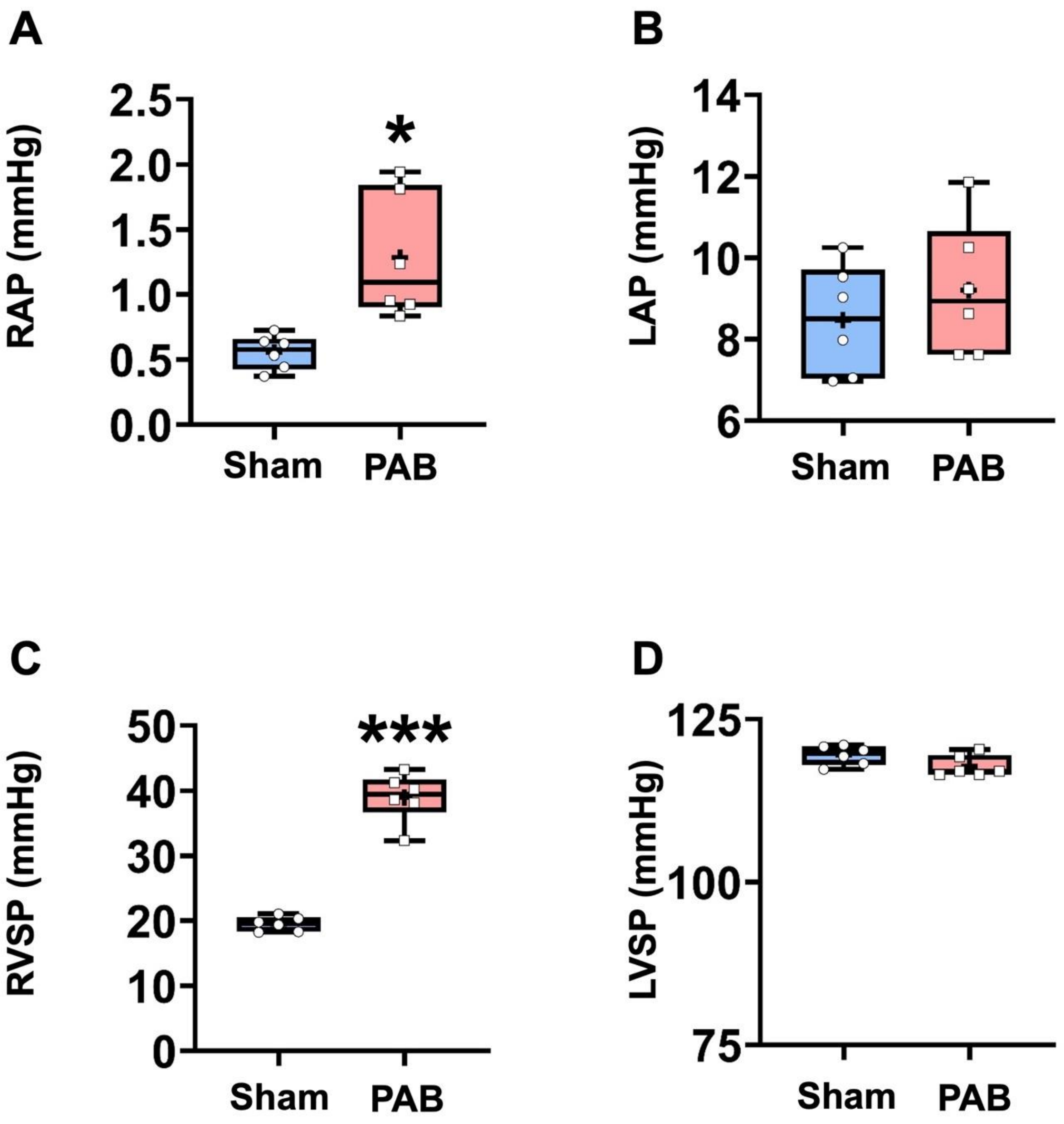

Figure S2. Hemodynamic Measurements. (**A**) Right atrial pressure (RAP) expressed in millimeter of mercury (mmHg), (**B**) left atrial pressure (LAP), (**C**) right ventricular systolic pressure, and (D) left ventricular systolic pressure (LVSP). *(Statistical analysis: Data were normally distributed was assessed by Shapiro-Wilk test and statistical difference between experimental groups was analyzed by Student's t-test. Each point represents an individual animal. n = 4 rats per group)*

Online Figure S3

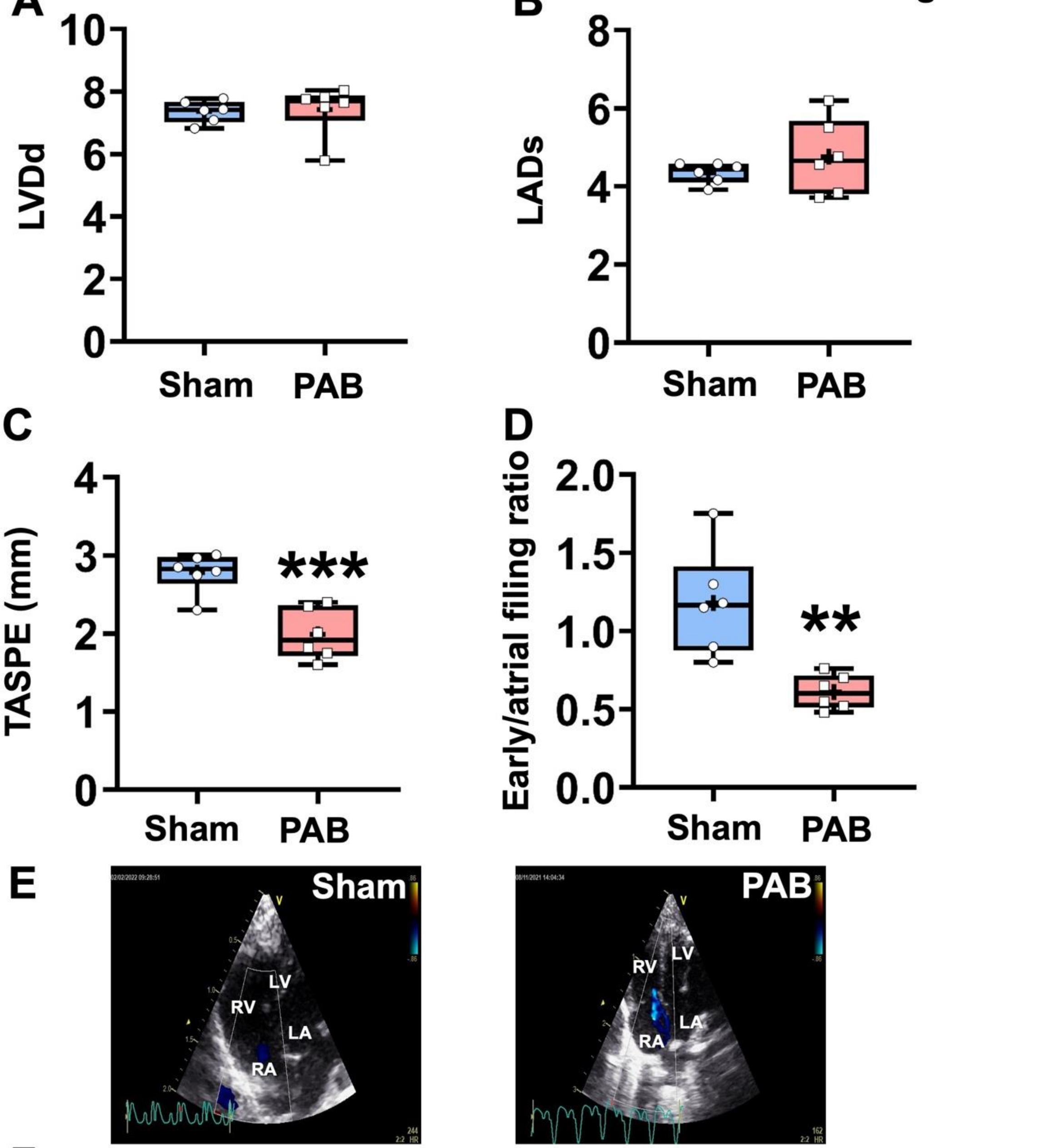

F

Tricuspid Regurgitation	Sham n = 6	PAB n = 6
Area (mm^2)	0	38.19 ± 8.5
Velocity (cm/s)	0	316.47 ± 30.57
G_{peak} (mmHg)	0	41.88 ± 7.64
G_{mean} (mmHg)	0	28.06 ± 4.59

Figure S3. Echocardiography. Graphs showing quantitative analyses of (**A**) LVDd, (**B**) LADs, (**C**) TAPSE, and (**D**) early/atrial filing ratio in sham and PAB rats. (**E**) Representative echocardiography images showing normal tricuspid valve closing in sham (left panel) and tricuspid regurgitation in PAB (right panel). (**F**) Parameters assessing tricuspid regurgitation in PAB, including the regurgitant area in mm^2, the regurgitant velocity in cm/s, the peak gradient (Gpeak) in mmHg, and the mean gradient (Gmean) in mmHg.

(Statistical analysis: A: non-normal distribution was assessed by Shapiro-Wilk test and statistical difference between experimental groups was analyzed by Mann-Whitney test. B, C, and D: normal distribution was assessed by Shapiro-Wilk test and statistical difference between experimental groups was analyzed by Student's T-test. Each point represents the level of expression from an individual animal. n=5 rats/group).

Online Figure S4

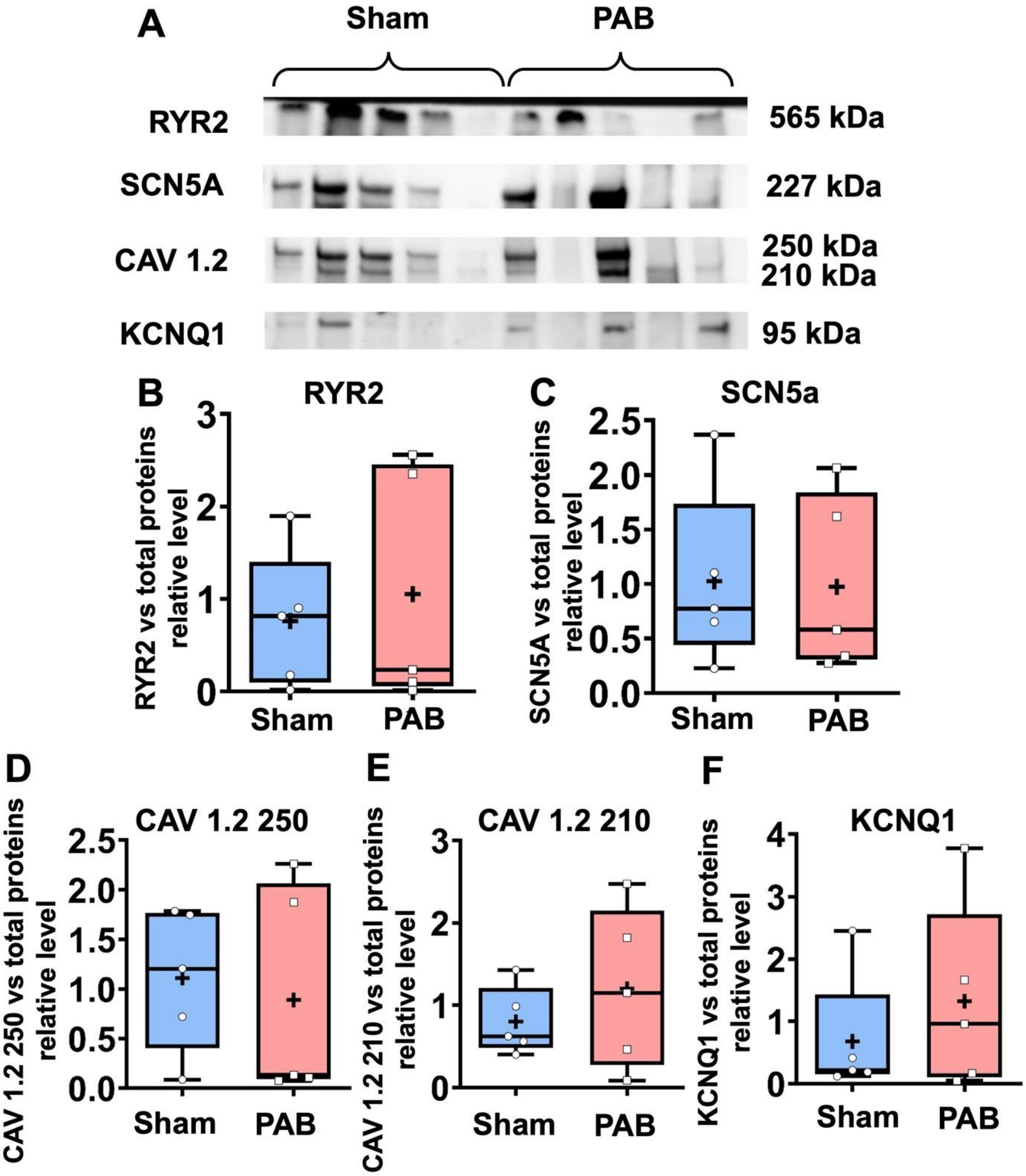

Figure S4. Protein expression of ion channel-related genes. Western blot analysis of RYR2 (**A** and **B**), SCN5A (**A** and **C**), CACNA1c (**A**, **D**, and **E**), and KCNQ1 (**A** and **F**). Images correspond to protein's bands from gels on which they were run compared to total proteins stained on blot. Uncropped membrane images and respective gels of total proteins stained on blot are available in Supplementary material online Figures S7 (RYR2), S8 (SCN5A), S9 (CACNA1c), and S10 (KCNQ1). *(Statistical analysis: B, D, and F: non-normal distribution was assessed by Shapiro-Wilk test and statistical difference between experimental groups was analyzed by y Mann-Whitney test. C: normal distribution analyzed by Student's T-test. Each point represents the level of expression from an individual animal. n=5 rats/group).*

Online Figure S5

Uncropped Western Blot Gels

SERCA2a
ThermoFisher, MA3-910, 110 kDa

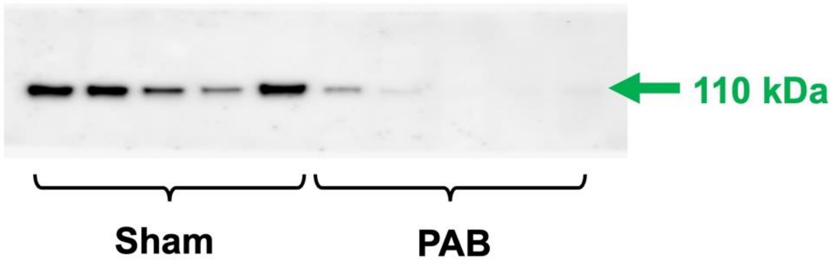

TOTAL PROTEINS ON BLOT

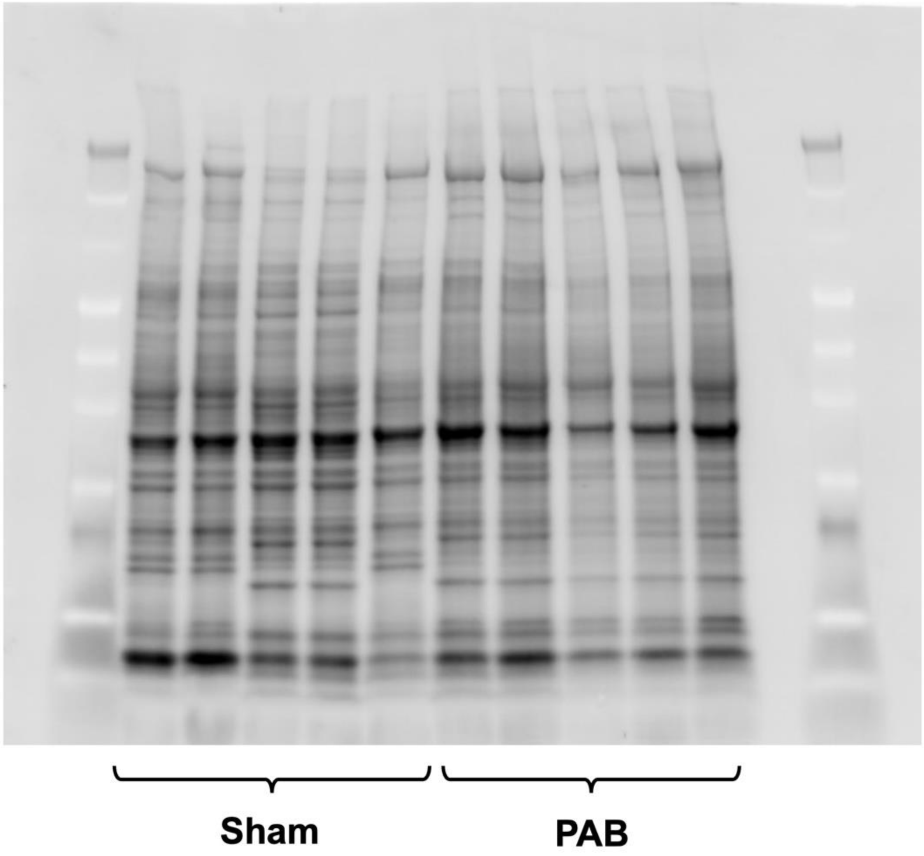

Online Figure S5. SERCA2a Original Western blot gel and Total proteins stained on Blot. (**Upper panel**) Uncropped western blot gel obtained from SERCA2a in RA from sham and PAB rats. Green arrow indicates the protein of interest and its molecular weight. (**Lower panel**) Image of total RA proteins tested by western blot from sham and PAB rats.

Online Figure S6

Uncropped Western Blot Gels

IL1β

Invitrogen, PA546956, 17 kDa

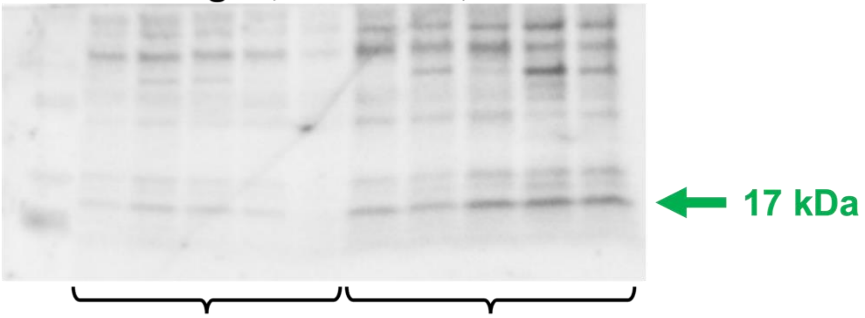

IL6

ThermoFisher Scientific, ARC0962, 26 kDa

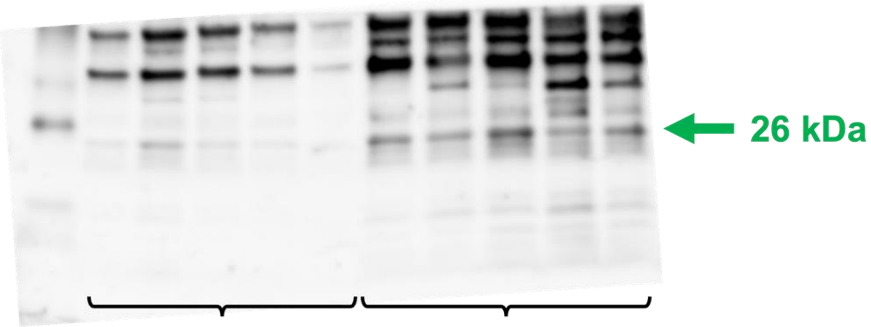

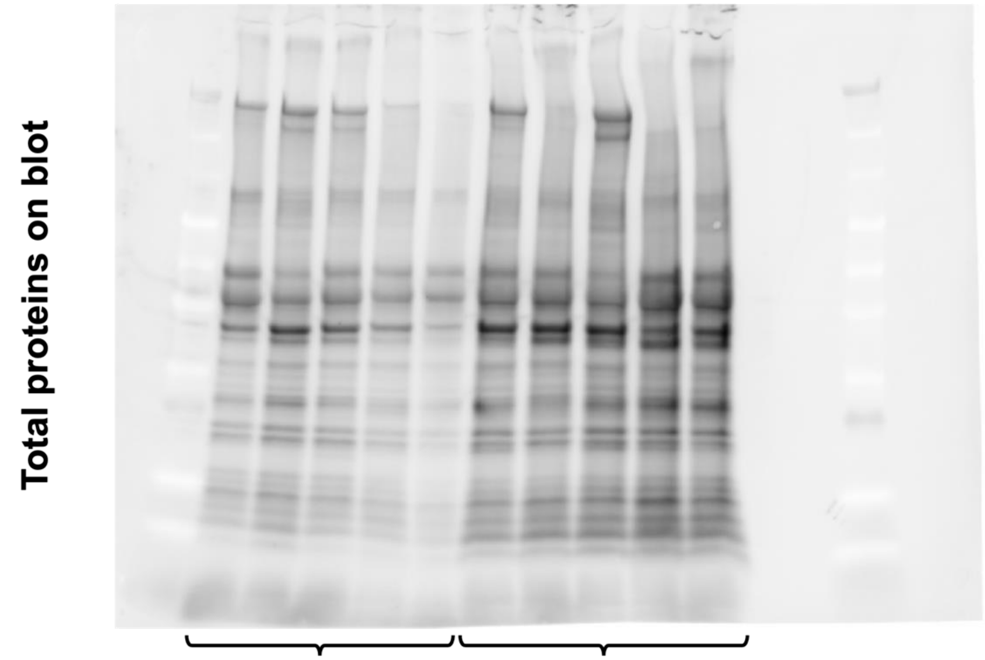

Online Figure S6. IL6 and IL1β Original Western blot gels and Total proteins stained on Blot. (**Upper panels**) Uncropped western blot gels obtained from IL6 and IL1β in RA from sham and PAB rats. Green arrow indicates the protein of interest and its molecular weight. (**Lower panel**) Image of total RA proteins tested by western blot from sham and PAB rats.

Online Figure S7

Uncropped Western Blot Gels

RYR2
ThermoFisher, MA3-916, 565 kDa

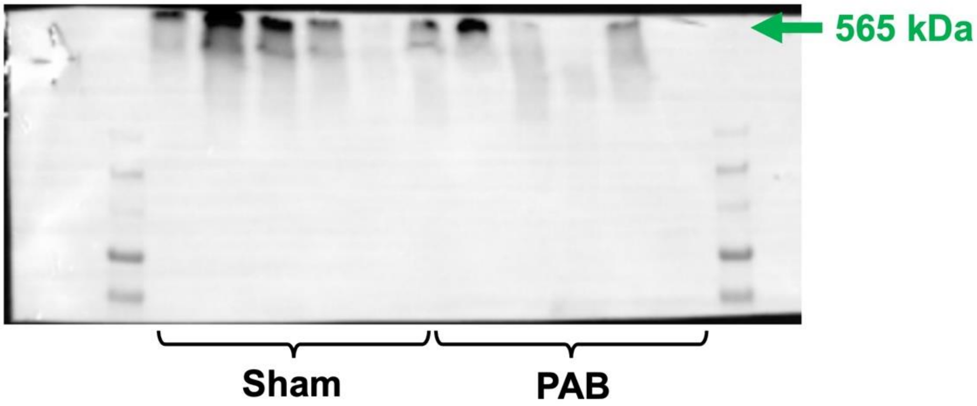

TOTAL PROTEINS ON BLOT

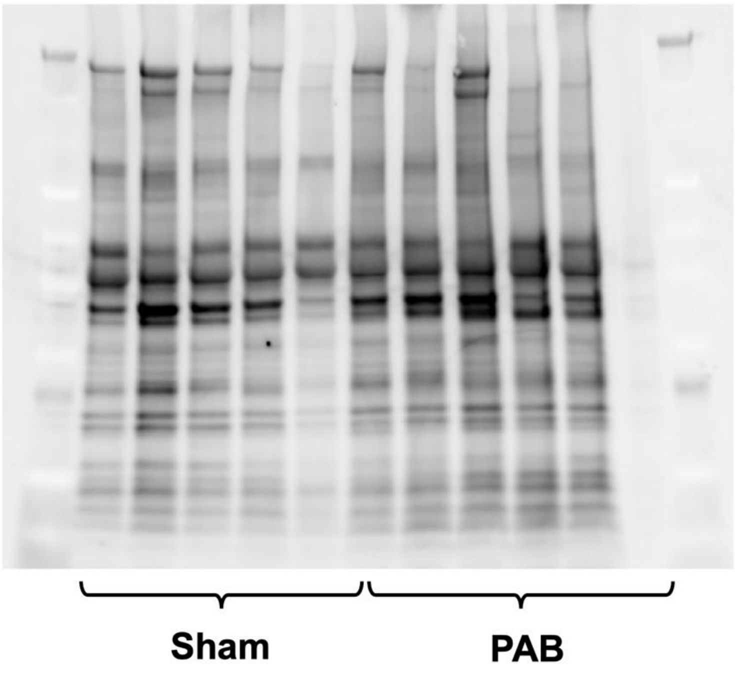

Figure S7. RYR2 Original Western blot gel and Total proteins stained on Blot. (**Upper panels**) Uncropped western blot gel obtained from RYR2 in RA from sham and PAB rats. Green arrow indicates the protein of interest and its molecular weight. (**Lower panel**) Image of total RA proteins tested by western blot from sham and PAB rats.

Online Figure S8

Uncropped Western Blot Gels

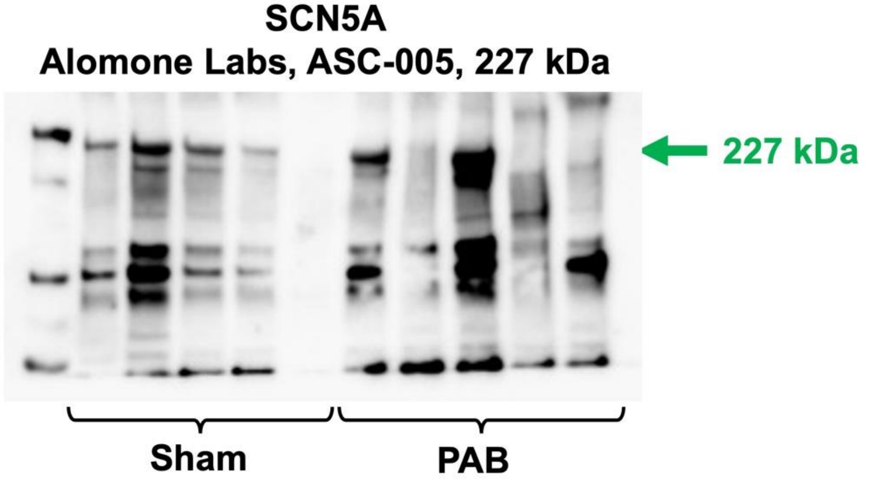

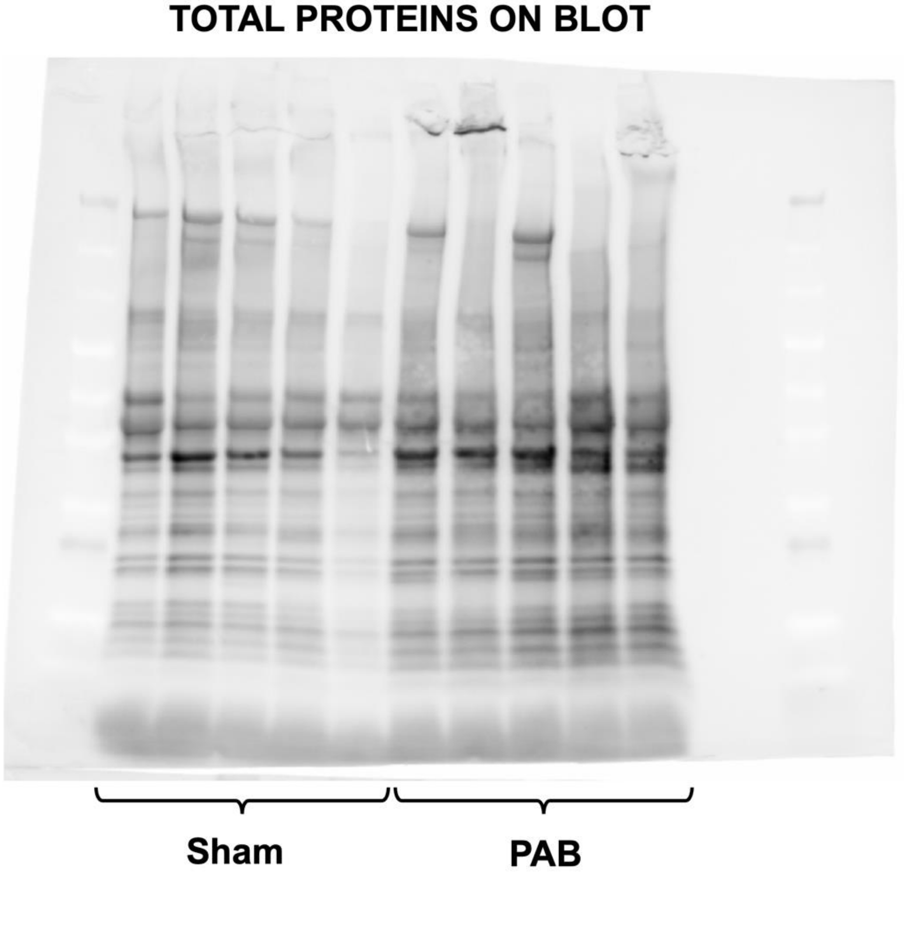

Figure S8. SCN5A Original Western blot gel and Total proteins stained on Blot. (**Upper panels**) Uncropped western blot gel obtained from SCN5A in RA from sham and PAB rats. Green arrow indicates the protein of interest and its molecular weight. (**Lower panel**) Image of total RA proteins tested by western blot from sham and PAB rats.

Online Figure S9

Uncropped Western Blot Gels

CAV 1.2
Alomone Labs, ACC-003, 250 and 210 kDa

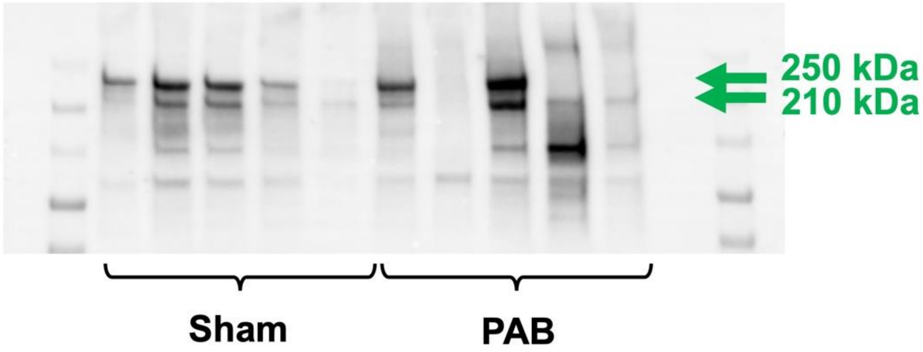

TOTAL PROTEINS ON BLOT

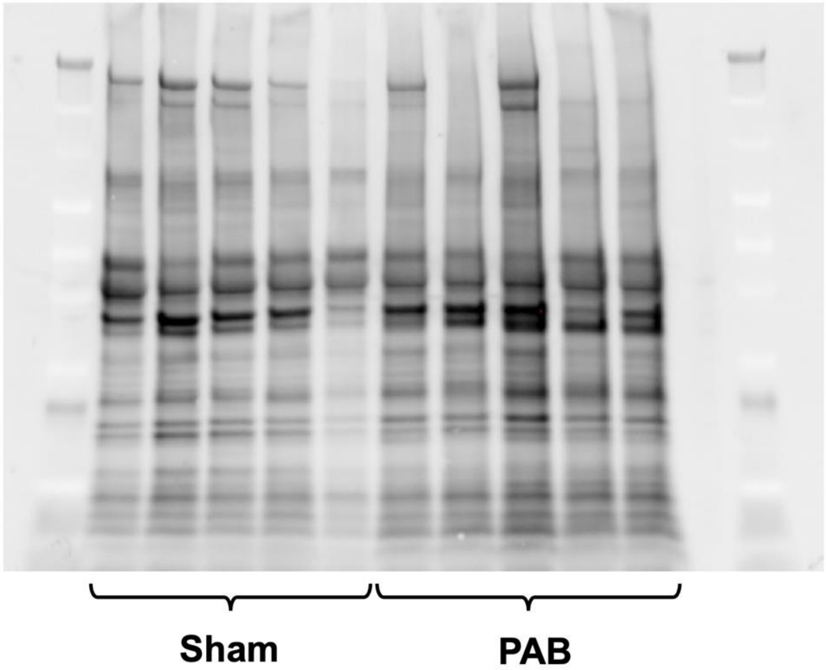

Figure S9. CACNA1c Original Western blot gel and Total proteins stained on Blot. (**Upper panels**) Uncropped western blot gel obtained from CACNA1c in RA from sham and PAB rats. Green arrows indicate the proteins of interest and their molecular weight. (**Lower panel**) Image of total RA proteins tested by western blot from sham and PAB rats.

Online Figure S10

Uncropped Western Blot Gels

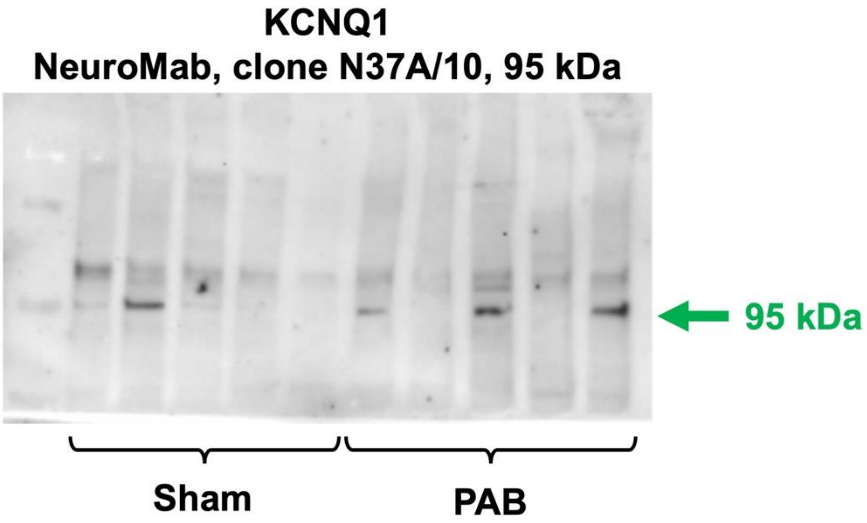

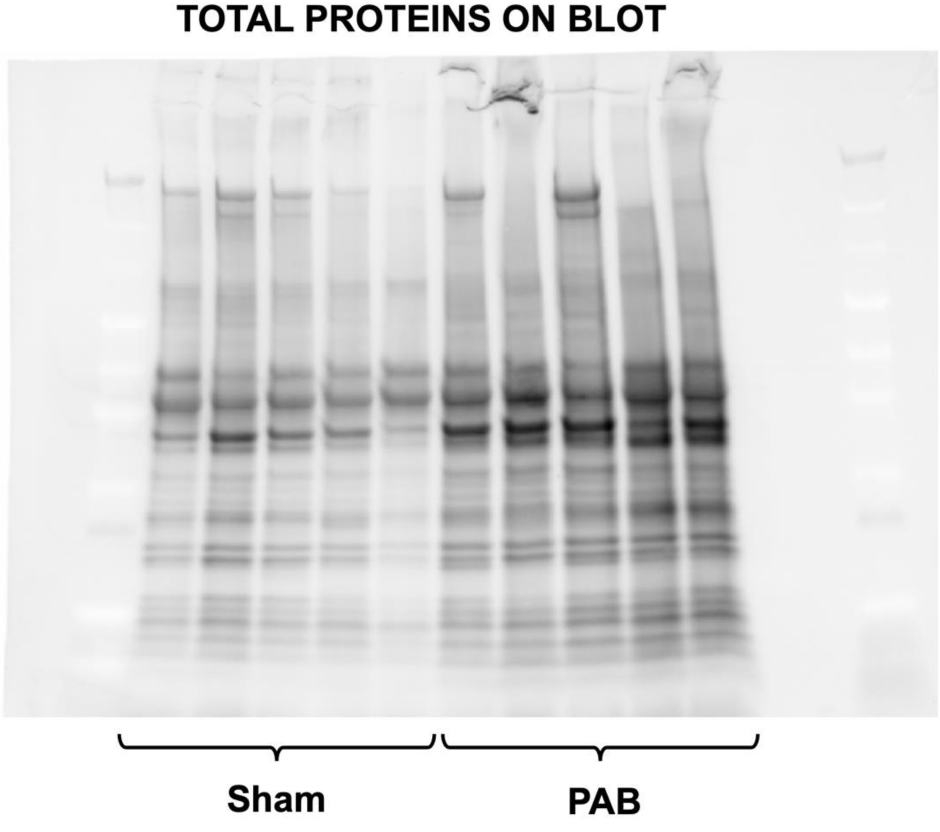

Figure S10. KCNQ1 Original Western blot gel and Total proteins stained on Blot. (**Upper panels**) Uncropped western blot gel obtained from KCNQ1 in RA from sham and PAB rats. Green arrow indicates the proteins of interest and their molecular weight. (**Lower panel**) Image of total RA proteins tested by western blot from sham and PAB rats.

Online Figure S11

Uncropped Western Blot Gels

BIN1
Novu Biologicals, NBP1-89102, 45 to 80 kDa

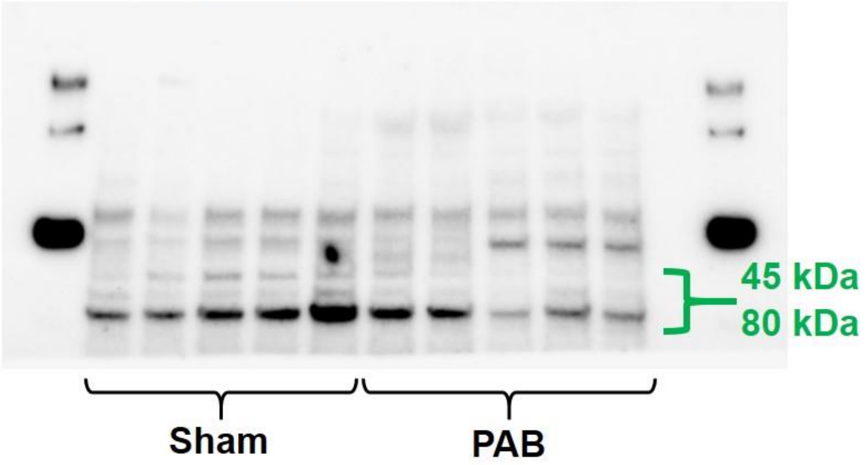

TOTAL PROTEINS ON BLOT

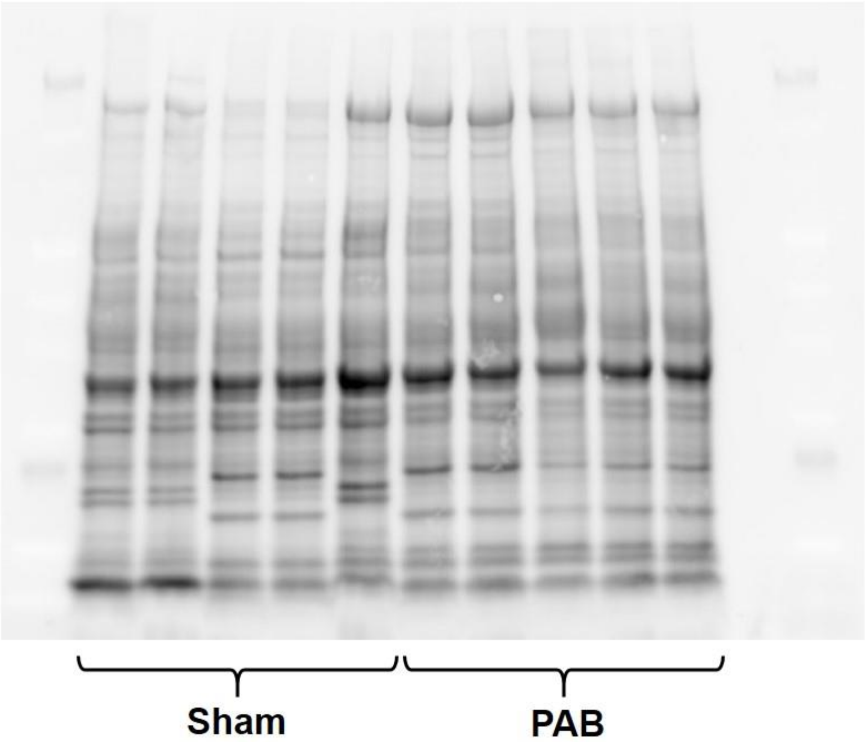

Figure S11. BIN1 Original Western blot gel and Total proteins stained on Blot.

(**Upper panels**) Uncropped western blot gel obtained from BIN1 in RA from sham and PAB rats. Green arrow indicates the proteins of interest and their molecular weight. (**Lower panel**) Image of total RA proteins tested by western blot from sham and PAB rats.

Online Figure S12

Uncropped Western Blot Gels

CAV3

Novus Biologicals, NBP3-16503, 17 kDa

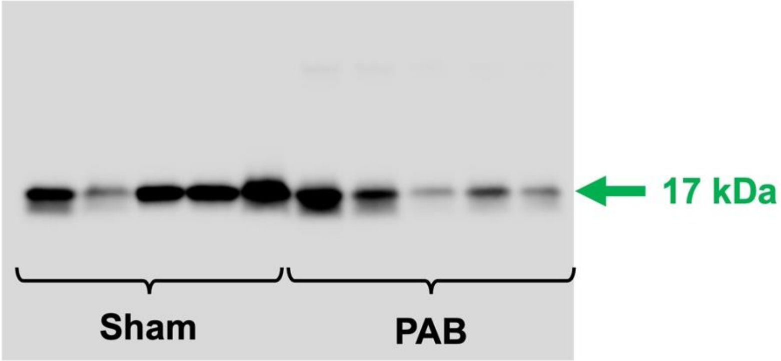

JPH2

ThermoFisher Scientific, PA5-141187, 74 kDa

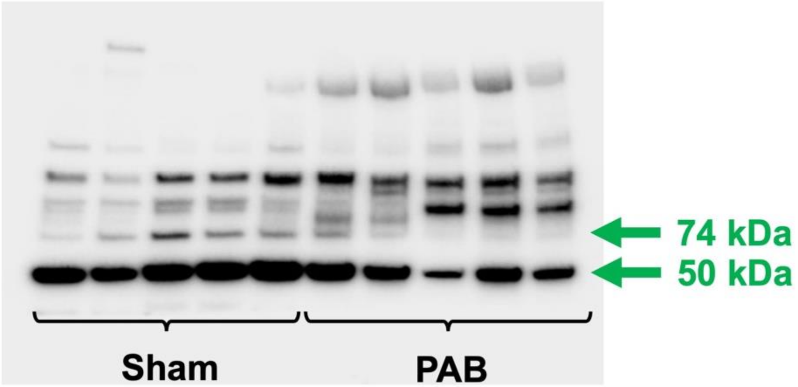

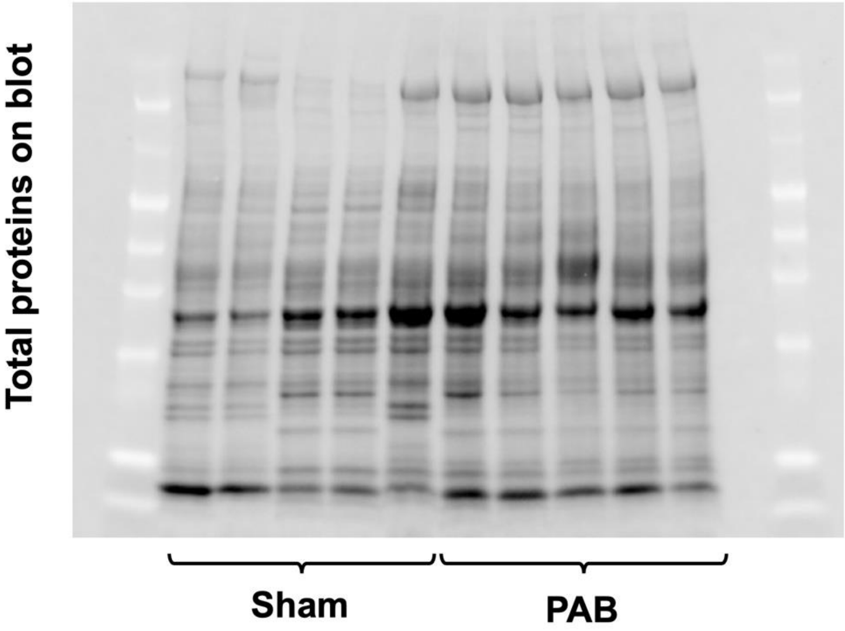

Figure S12. CAV3 and JPH2 Original Western blot gels and Total proteins stained on Blot. (**Upper panels**) Uncropped western blot gels obtained from CAV3 and JPH2 in RA from sham and PAB rats. Green arrow indicates the protein of interest and its molecular weight. (**Lower panel**) Image of total RA proteins tested by western blot from sham and PAB rats.
